# The perils of omitting omissions when modeling evidence accumulation

**DOI:** 10.1371/journal.pcbi.1014667

**Published:** 2026-08-21

**Authors:** Xiamin Leng, Alexander Fengler, Amitai Shenhav, Michael J. Frank

**Affiliations:** 1 Department of Psychology, University of California, Berkeley, California, United States of America; 2 Department of Cognitive and Psychological Sciences, Brown University, Providence, Rhode Island, United States of America; 3 Helen Wills Neuroscience Institute, University of California, Berkeley, California, United States of America; 4 Carney Institute of Brain Science, Brown University, Providence, Rhode Island, United States of America; University of Minnesota, UNITED STATES OF AMERICA

## Abstract

Response deadlines are commonly imposed in decision-making research to incentivize speedy decisions and sustained attention. This procedure frequently leads to a proportion of trials in which no response is made, and these omissions are often simply removed from the data during analysis. Here we show that this seemingly trivial assumption is in fact quite consequential for parameter estimation. We propose that omissions should instead be treated as observations to inform inference of the underlying generative process that led to their occurrence. Using new tools from likelihood-free inference applicable to a broad class of sequential sampling models (SSMs), we enable fast computation of omission probability without explicit integration, and clarify the degree to which omitting omissions – even in seemingly benign settings – can lead researchers astray. We explore this phenomenon in the setting of SSMs with constant and time-varying boundaries, and show that parameter recovery is improved by incorporating a model of omission probability. We show that the benefits of modeling omission probability are distinct from benefits gained from past approaches of modeling attentional lapses to account for these omissions. Our findings shine a light on the consequences of omitting omission in modeling choice behavior with SSMs, and demonstrate how joint modeling of observed and omitted data can improve parameter inference and therefore the reliability of downstream scientific conclusions.

## Author summary

Many studies of human decision-making use tasks that impose time limits, but researchers often ignore trials in which participants fail to respond before the deadline. This paper shows that such “omissions” are more informative than they might appear—and that leaving them out can lead to misleading conclusions. The authors demonstrate that ignoring omissions distorts the estimated parameters that describe how people accumulate evidence and make choices under time pressure. To address this, they develop a new computational approach that models both responses and omissions together. Using modern machine-learning tools, specifically Likelihood Approximation Networks (LANs) combined with a new Omission Probability Network (OPN), the method efficiently estimates how likely omissions are to occur under different model settings. Across a range of decision-making models and conditions, this joint modeling approach greatly improves the accuracy and reliability of parameter estimation, even when omissions are rare. The work highlights how small analytic shortcuts—like excluding no-response trials—can meaningfully affect scientific inference and proposes a practical solution implemented in an open-source software package, making it easier for researchers to include omissions in their models and produce more trustworthy conclusions about the mechanisms of human decision-making.

## Introduction

Joint modeling of choices and response times is a core methodological approach for unveiling the dynamics of decision making. An important class of cognitive process models used for this purpose is known as Sequential Sampling Models (SSM; [1–4]). Variants of such SSMs are routinely used to dissect choice behavior into interpretable components. These models conceptualize decision making as *boundary crossings* of a random particle with drift. The momentary position of the particle represents a state of *evidence* of the system, and the momentary distance between choice boundaries represents the amount of *accumulated evidence* required to commit to a choice (or *decision threshold*). Crossing the boundary implies a choice and a corresponding response time on a given trial. Many studies have shown that this class of models provides a coherent account of choice proportions and their RT distributions across a range of paradigms whereby, for example, the drift rate is related to the strength of evidence in the signal (high rates lead to more accurate and faster RTs) and the boundary separation modulates the speed-accuracy tradeoff (higher bounds lead to slower but more accurate responding; [3,5]). However, not every decision process ends in a boundary crossing, leaving open questions about how to treat trials without a recorded choice.

Based on normative analyses, extensions of SSM models allow decision boundaries to *collapse* over time, such that the amount of evidence needed to commit to a choice is lower as time evolves [6]. This dynamic is believed to be especially beneficial for decision-making studies that involve a choice deadline: as the deadline approaches, the decision-maker should commit to the choice that has the most evidence even if they are not as confident about that choice as they would like to be given sufficient time [7–9]. In addition to deadlines, boundary dynamics are also sensitive to properties of the choice options, such as those that lead to more difficult decisions [10] or otherwise generate decision conflict [11–15]. Such collapsing boundaries then serve as an “urgency-signal” for the subject to force a decision in service of a global objective function imposed by the experiment design [16,17]. Thus, estimating how decision parameters, including boundaries, vary according to cognitive and neural factors remains an active area of research that is informed by rigorous parameter estimation.

In practice, while deadlines have been used to induce speedy decision-making and manipulate time pressure, especially when studying the underlying neural dynamics [18–22], it is often inevitable to have trials for which no response is given (e.g., omissions; see Table A1 in S1 File for examples; [18,22–25]). When analyzing such data, it is commonplace to exclude such omissions and focus only on the trials with committed responses [18,21,26–28]. The implicit assumption has been that excluding these omitted trials will not significantly impact parameter estimation, and therefore downstream conclusions, but the extent to which this is the case typically goes untested. It is crucial to recognize that the profound implications of omitted responses and data truncation on reaction time distributions have been raised in [29]. A more practical limitation has been that a majority of SSMs are only able to make predictions about committed responses, preventing studies from using these models to make inferences about omissions.

Recently, evidence accumulation models have sought to incorporate omissions [30,31]. While it can be easy to derive omission probability from the cumulative distribution function of responses for some cases that permit analytical calculations [31], it becomes computationally difficult in many cases, even for simple variations of canonical cognitive process models. Such “intractable” models, however, are becoming more popular as researchers are interested in accounting for neural nonlinearities and other time-varying processes during decision making [11,16]. Numerical and simulation-based methods have been implemented to accommodate omissions [32,33]. However, the availability of these methods does not equate to a low practical burden. The numerical approach [32] requires extensive computation to obtain probability density function. Simulation-based inference improves the calculation [30], but does not handle dataset with hierarchical design naturally. Existing packages (for example, HDDM, SBI, EMC2; [34–36]) for evidence accumulation models generally lack native support for non-responses. Therefore, researchers might have to handle simulations, mixture likelihoods, and hierarchical structures to account for omissions. All of these could increase the burden of model usage, frequently biasing researchers toward the heuristic approach of ignoring omissions entirely.

This paper addresses three questions: 1) What are the consequences of omitting omissions when inferring decision dynamics? 2) How to efficiently incorporate omissions into parameter estimation? 3) To what extent does including omission improve the identification of model parameters? Past studies [31,37] estimated omission probabilities by integrating the post-deadline proportion of probability density function. However, these methods can be computationally expensive even for SSMs with analytic likelihood function, and this issue is especially pernicious for a broad class of SSMs that don’t have analytic likelihood functions. It would be beneficial to incorporate estimation of omission probability into sequential sampling models in an efficient way. The introduction of Likelihood Approximation Networks (LANs; [4,38]) has enabled researchers to fit a broad class of such models by pretraining neural networks to approximate likelihood functions, comprising a form of amortized Bayesian inference [39,40]. Building on this advancement, we extended the LAN framework to empirically investigate the vulnerability to misleading conclusions that arise when omissions are ignored. Rather than computing integrals analytically, we trained a second type of network, dubbed Omission Probability Network (OPN), directly on the omission probabilities, given SSM parameters and a deadline. We show with a series of numerical experiments that parameter estimation (and therefore conclusions about mechanisms driving cross-condition differences in behavior) can be severely affected if omissions are not explicitly modeled, even when such omissions are relatively rare. We then demonstrated that this limitation can be eliminated by joint modeling of observed data and omission using our LAN + OPN approach. We validate this conclusion with three different types of decision boundary (constant, linearly collapsing, and nonlinearly collapsing), and show that our model can distinguish between omissions arising from evidence accumulation and those arising from attentional lapses.

## Methods

### Sequential sampling models

In this paper, we begin with the drift diffusion model (DDM) as the basis from which we derive two additional variations as test-beds for our numerical experiments: the ANGLE and WEIBULL models. The DDM is a basic sequential sampling model (SSM), ubiquitously applied with a long history in cognitive science [2]. The standard DDM is formulated as


$$\begin{array}{c} dX_{t}=vdt+cdW,\;X_{\text{0}}=\text{2}*z*a \end{array}$$

by setting $f_{bound}^{DDM}(t)=a$, a fixed boundary value, v the drift rate, cdW, a Gaussian noise scaling over time, and z, the bias at the starting point of the diffusion process. A decision is made when $X_{t}$ reaches lower (0) or upper boundary (2*a). It is common to add a non-decision time τ to the model as a means to collect residual time spent on perceptual or motor processes not related to the choice process. Here, without loss of generality, we set z at 0.5 (e.g., equal distance to upper and lower boundary) and τ at 0.3 s.

DDMs with collapsing bounds have been used in particular for situations with response deadlines [41,42], as a rational decision maker can force themselves to decide with incomplete evidence, especially if rational under a global objective function to maximize reward of an entire block of trials [6]. We will call a DDM with a linearly collapsing boundary the ANGLE model, and define the boundary as follows,


$$\begin{array}{c} f_{bound}^{ANGLE}(t)=a-t\times \frac{sin(\theta )}{cos(\theta )} \end{array}$$

**Fig 1:**
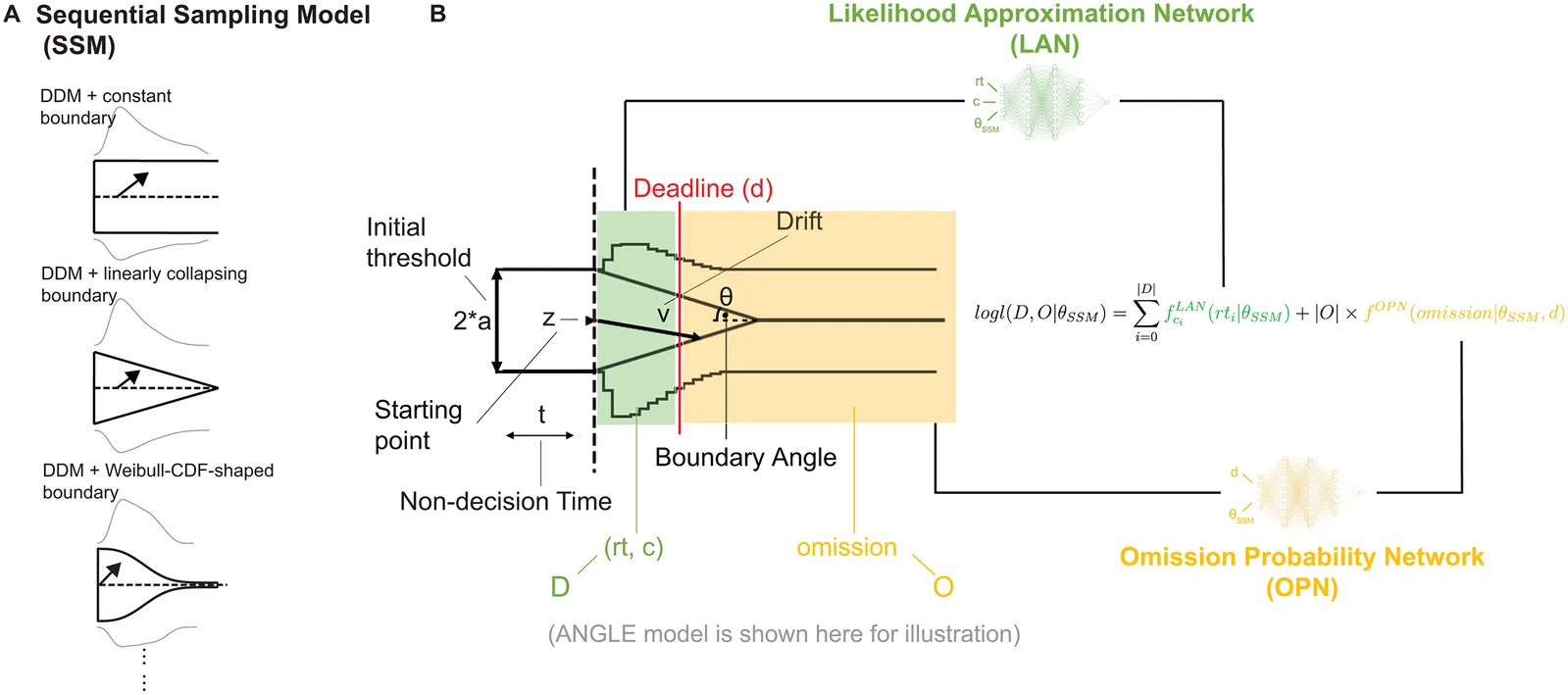
Illustration of the log-likelihood formulation with the help of a LAN and an OPN. **A)** We consider drift diffusion model (DDM), a representative sequential sampling model (SSM), with constant, linearly collapsing, and nonlinearly collapsing boundary shapes. **B)** In our proposed framework, the OPN provides a function $f^{OPN}$for the log-likelihood of omissions, given a deadline setting. We simply multiply the log-likelihood of omissions by the number of omitted trials, which is enough for the numerical experiments introduced here. In principle, trial-wise deadlines can be incorporated at no extra cost. The LAN provides the function $f^{LAN}$for the log-likelihood of observing a given (rt,c) response time and choice pair prior to the deadline. Both networks take $\theta_{SSM}$-the parameters of the underlying sequential sampling model. The LANs input adds to this the observed pair (rt,c), while the OPN adds to this the deadline d. The general framework is applicable to a large class of sequential sampling models **(A)**, of which the model shown here is simply a concrete example based on ANGLE model **(B)**.

Here θ indicates the boundary collapsing angle with allowable range in the interval (0,π/2). The linearly collapsing boundary serves to represent the concept of urgency in decision making, which, as mentioned, has been proposed as theoretically relevant repeatedly. Our third alternative, which we call the WEIBULL model, takes as boundary shape the nonlinear Weibull function [16]:


$$\begin{array}{c} f_{bound}^{Weibull}(t)=a\times exp(-{\left(\frac{t}{\beta }\right)}^{\alpha }) \end{array}$$

where α is the shape parameter and β is the scale parameter. Our analysis in this paper will cover the DDM with all three types of boundary shape (constant, ANGLE, WEIBULL; Fig 1A). For the sake of simplicity, we only consider models without inter-trial variability of drift rate, starting point bias and non-decision time. We expect that our findings can be generalized to those models.

### Likelihood

When there is no response deadline, an SSM will eventually produce a response on each trial, and there will be no omissions. The likelihoods for observed responses based on SSMs with two alternatives are described by a set of two defective distributions (one for each choices, c=1 and c=−1), $f_{c}(t;\theta_{SSM})$, where,


$$\begin{array}{c} \int_{\text{0}}^{+\infty }f_{\text{1}}(t|\theta_{SSM})dt+\int_{\text{0}}^{+\infty }f_{-\text{1}}(t|\theta_{SSM})dt=\text{1} \end{array}$$

and $\theta_{SSM}$ represents the set of parameters for our given SSM. However, when a response deadline dexist, only responses before the deadline will be observed, and the rest of trials will be treated as omissions. Here we are specifically interested in the influence of inclusion and exclusion of omissions on parameter recovery, and thus two types of data need to be considered in the model.

1. Observed data D: $d_{i}=({rt}_{i},c_{i})$ where ${rt}_{i}\leq d$ and both RT and choice are recorded.
2. Omission O: $d_{i}=({rt}_{i},c_{i})$ where ${rt}_{i}>d$ and neither RT or choice is recorded. Instead, the trial is masked with a boolean value indicating omission.

For observed data points we use our functions $f_{c}(t;\theta_{SSM})$ above. For omissions, however, we need to compute the following integral,


$$\begin{array}{c} p(omission|\theta_{SSM})=\int_{d}^{+\infty }f_{\text{1}}(t|\theta_{SSM})dt+\int_{d}^{+\infty }f_{-\text{1}}(t|\theta_{SSM})dt \end{array}$$

In this integral, d indicates the response deadline imposed by experiment and is treated as a known parameter. Since these integrals are not typically considered or available in relevant software packages for SSM parameter estimation researchers choose to treat omissions as missing data, and thus perform inference using only the probability density functions for observed data described above. In this paper, we used 1.25 s as the deadline, but the general pattern persists with different deadline settings when omission rate is similar (see Fig A3 in S1 File).

Here we address this problem by extending the framework of likelihood approximation networks (LAN; [38]), to enable fast inference of arbitrary SSMs while including the likelihood of omissions explicitly. To be consistent across our analyses and without loss of generality, we use the LAN as the likelihood for all observed responses from SSMs, even though analytical likelihoods are available for the standard DDM case. We previously showed that the LAN provides an excellent approximation of the DDM likelihood and can even be more efficient in computation time than the analytical likelihood, because it can be parallelized over trials. Thus, all of our comparisons to the “LAN only” approach would apply equally for analytic likelihoods; the main results of this paper should thus be taken to showcase the utility of OPNs, not to judge the adequacy or inadequacy of LANs *per se*. In particular, we use a simulator that returns the probability of missing the deadline, which can then be used to train an OPN to estimate this probability based on SSM parameters and response deadline. Hence, we train two LANs for each model, one for the function $f_{c}^{LAN}(t|\theta_{SSM})$ that will, and one for the function $f^{OPN}(omission|\theta_{SSM})$. The log-likelihood of a given dataset (D,O), is then computed as,


$$\begin{array}{c} ogl(D,O|\theta_{SSM},d)=\sum_{i=\text{0}}^{\left|D\right|}f_{c_{i}}^{LAN}({rt}_{i}|\theta_{SSM})+|O|\times f^{OPN}(omission|\theta_{SSM},d) \end{array}$$

this combined approach enables estimation of $\theta_{SSM}$ via fitting observed choice data and omissions jointly (Fig 1B).

### Inclusion of lapse distribution

While our primary analysis assumes that all choices are generated from a single SSM parameterization, empirically, it is also possible that some proportion of responses and omissions reflect attentional lapses. To test how our approach performs in this case, we followed the setup of contaminant process in [43] and modeled lapse process as a uniform distribution over a wide range of time T (5s in our analysis) and assuming that lapses generate random choices (in this case, p = 0.5 for each choice option):


$$\begin{array}{c} f^{Lapse}(t)=\frac{\text{1}}{\text{2}\times T}\;for\;t\in [\text{0},T] \end{array}$$

In this case, omissions from the lapse distribution simply means a sample with rt>d drawn from lapse distribution.

With this setting of lapse distribution, both observed data and omissions are drawn from a mixed distribution of SSM and lapse. The proportion of lapse is quantified as $p_{lapse}$ and thus the weight of SSM is $\text{1}-p_{lapse}$. The joint likelihood of observed data and omission is


$$\begin{array}{c} ikelihood=(\text{1}-p_{lapse})\times {likelihood}_{SSM}+p_{lapse}\times f^{Lapse} \end{array}$$

where ${likelihood}_{SSM}$ is based on Equation (6). This likelihood enables joint estimation of SSM parameters as well as the lapse proportion, whether considering omission or not.

### Training of LAN and OPN

The training and setup for LANs in the current paper follow exactly the recipe in the foundational methods paper (see Part 2 in S1 File for a brief summary; [38]). For OPN, we followed the same procedure with a difference in the training data structure: inputs of OPN include model parameters and deadline and each combination of input has one simulated omission rate value (Fig 1). No reaction time distributions need to be constructed for the omission probability outputs that serve OPN training.

### Simulation and inference

All models were simulated with a fixed unbiased starting point (coded as `z` = 0.5) and a fixed non-decision time (t). We generated 1000 trials based on the evidence accumulation process with a time step of 0.001s (see Table A2 in S1 File for details of model parameters). Trials with reaction time longer than predefined deadline are masked. All models were fit via MCMC, where we estimated the posterior distributions using the No U-Turn Sampler (NUTS; [44]). We sampled 4 chains from the posterior distributions in each model with 1000 warmup samples followed by 1000 actual samples. In each model, we fixed the value of starting point bias and inferred non-decision time, drift rate, boundary parameters, and probability of lapse (see Table A3 in S1 File for details of prior distributions).

## Results

### Influence of omitting omissions for DDM with constant boundary

**Fig 2:**
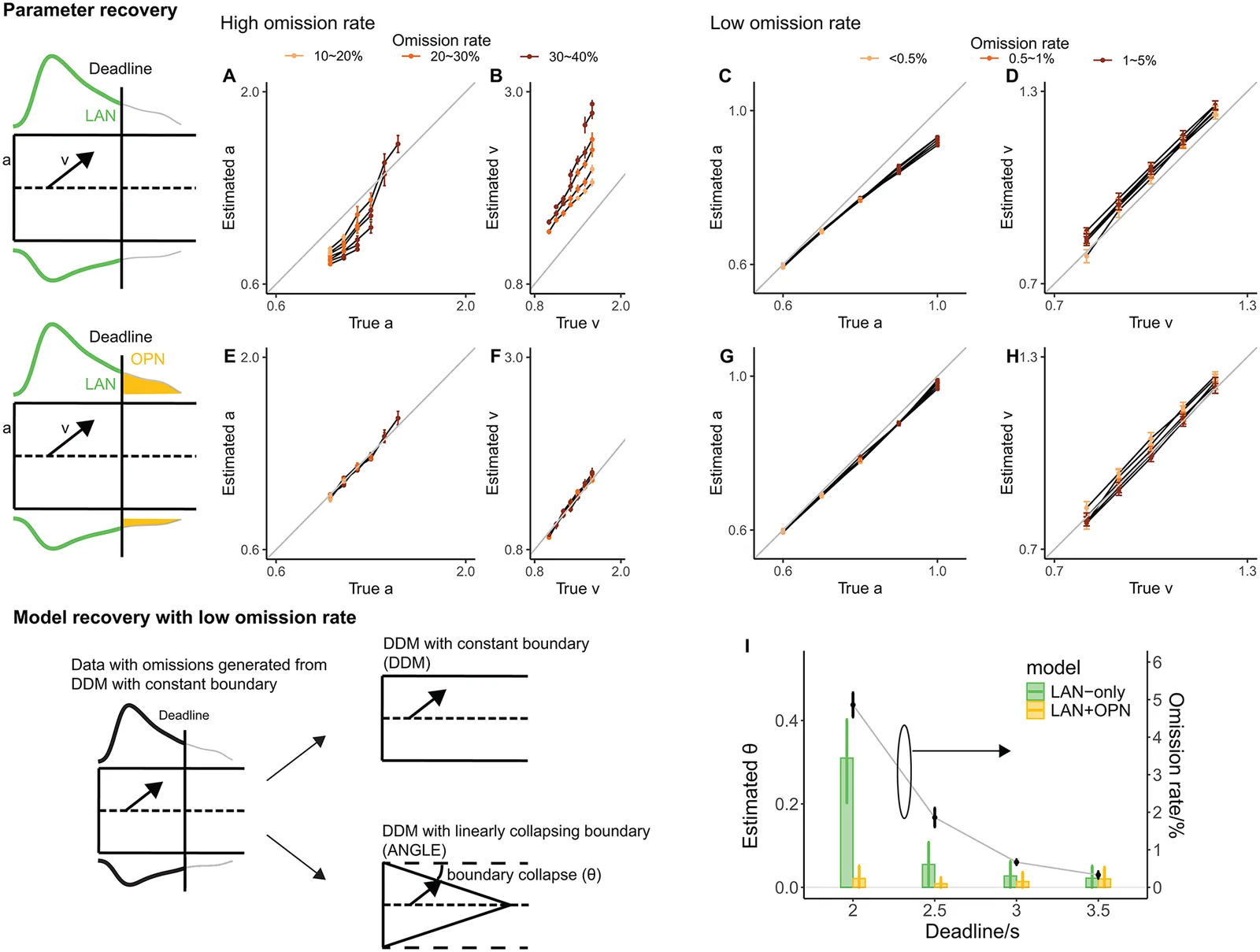
Parameter recovery performance for DDM model with constant threshold, when either ignoring omissions (e.g., only considering likelihood of responses before deadline; LAN-only model; A-D) or including them via the omission probability network as explained in Fig 1 (e.g., including integral of post-deadline likelihood; LAN + OPN model; E-H). When omission exists, the LAN-only approach underestimates response threshold a **(A)** and overestimates drift rate v **(B)**. This bias emerges even when omission rate is lower than 5% but higher than 1% **(C, D)**. With the LAN + OPN approach, both threshold a **(E, G)** and drift rate v **(F, H)** are properly recovered. Model recovery for DDM model with constant threshold **(I)**. When omissions are ignored (LAN-only model), the posterior distribution of boundary collapse deviates from ground truth (zero) even when omission rates are less than 5%, while LAN + OPN model can properly recover the constant boundary. Error bars refer to 95% confidence intervals of mean posteriors across multiple synthetic data sets from the same parameter configuration. Gray lines represent identity lines.

A DDM with a constant decision boundary—where evidence accumulates over time until it reaches a fixed threshold—typically predicts response time (RT) distributions with a long tail, meaning that a non-negligible portion of responses would occur only after very long delays. Therefore, it also predicts that a considerable fraction of these expected responses will be omitted under a realistic response deadline. Without compensation for these omissions, constant-boundary DDMs that are fit to the remainder of the data will estimate the threshold a as being lower than it actually is (Fig 2A), even when omission rate is as low as 5% (Fig 2C). Moreover, fitting with only a LAN (in this case, equivalent to fitting the analytical likelihood function for the DDM but ignoring omissions) also overestimates drift rate v and recovers larger than accurate drift rate parameters for higher omission rates (Fig 2B), and the bias emerges with relatively low omission rate (Fig 2D). These misestimations arise because the model cannot penalize omissions through the likelihood term and therefore prioritizes parameters supported by faster responses (e.g., lower threshold and higher drift rate). In contrast, when we jointly model observed choices and omission with the LAN + OPN approach, we obtained reliable parameter recovery for various combinations of (a,v) with different levels of omission rate (Fig 2E-2H). Additional simulations based on an independent race model [45] confirm that omitting omission can similarly bias estimation of parameters in a wider range of evidence accumulation model with a constant boundary even when omission rate is low (~5%; see Fig A1 in S1 File).

As the DDM with constant boundary does not consider urgency and boundary collapse, a more prominent concern with omitting omission is whether the remaining observed data supports a DDM model with collapsing boundary, even when the underlying generative process follows a DDM with constant boundary. To demonstrate this concern, we generated data from DDM with constant boundary and applied deadline, and fit the data with a DDM with linearly collapsing boundary (Fig 2I). LAN-only model does not strongly support a DDM with a linearly collapsing boundary (e.g., ANGLE model) when the omission rate is very low (~1%). However, as the omission rate increases (~5%), the posterior sample of boundary collapse significantly deviates from 0. By considering the omissions in the model, we found that DDM with constant boundary can be correctly identified from the data, even when omission rate is high (~5%).

### Influence of omitting omission for DDM with linearly collapsing boundary

**Fig 3:**
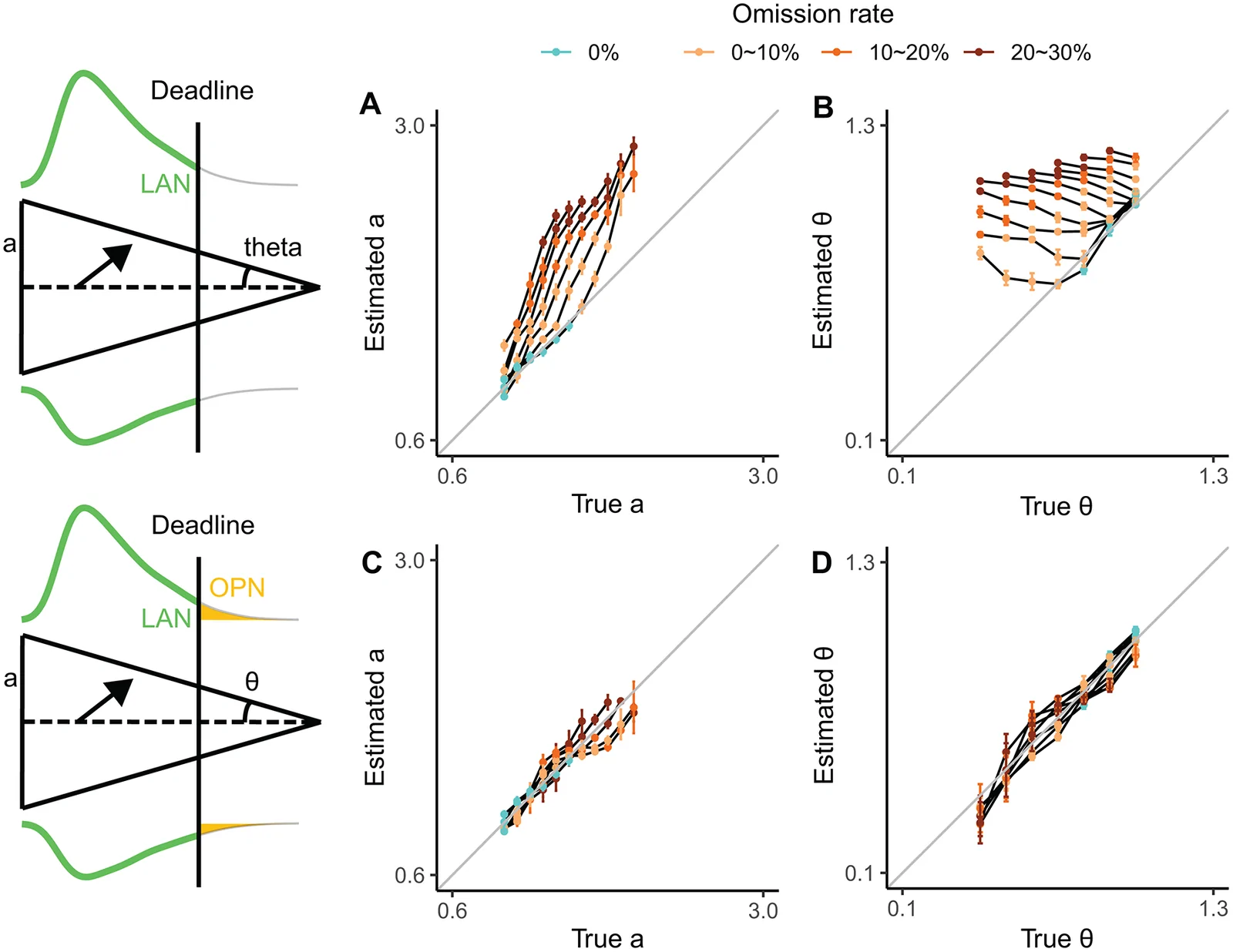
Parameter recovery performance for DDM model with linearly collapsing boundary, with either the LAN-only approach (A-B) or the LAN + OPN approach (C-D). When omission exists, LAN-only model overestimates both initial response threshold a **(A)** and angle of collapsing boundary θ **(B)**. With the LAN + OPN-based approach, both initial threshold a **(C)** and angle of collapsing boundary θ **(D)** are properly recovered. Error bars refer to 95% confidence interval of mean posteriors across multiple synthetic data sets from the same parameter configuration. Gray lines represent identity lines. Black lines indicate same true θ value in **A** and **C**, and same true a value in **B** and **D**.

While we have confirmed that the existence of omissions can bias parameter estimation when a model ignores omissions, as noted above, the constant bound DDM may not be the ideal model with response deadline, as it does not take urgency into consideration [7,41]. A DDM with a collapsing boundary may therefore be less vulnerable to the existence of omissions because it better captures distributions of responses under time pressure (e.g., shorter reaction time responses). We begin by examining a version of the collapsing boundary model that collapses linearly, using the ANGLE model which we have previously shown can be efficiently estimated with a LAN [38]. Here we primarily focused on the recovery of boundary parameters (a,θ). When there is no omission in the simulated data, the LAN-only model adequately recovers r the boundary parameters (blue dots in Fig 3). But when omissions exist in the data (reddish dots in Fig 3), recovered values of a and θ are overestimated when only a LAN is used. Specifically, when omission is not considered, the LAN-only model inferred more aggressive collapsing boundary than the generative process, with a overestimated especially when true a is high and θ overestimated when true θ is low (Fig 3A and 3B), as this leads to minimal omission probability. While the bias increases as omission rate increases (e.g., more missing data), it is worth noting that significant bias emerges even when omission rates are lower than 5%. In contrast, using the LAN + OPN approach, we obtained reliable parameter recovery for various combinations of (a,θ) and different levels of omission rate (Fig 3C-3D), as the inclusion of omissions informs the model of the fact that the actual response boundary does not collapse fast enough to eliminate omissions.

### Modeling omissions better estimates experimental condition effects on decision boundary: A synthetic demonstration

**Fig 4:**
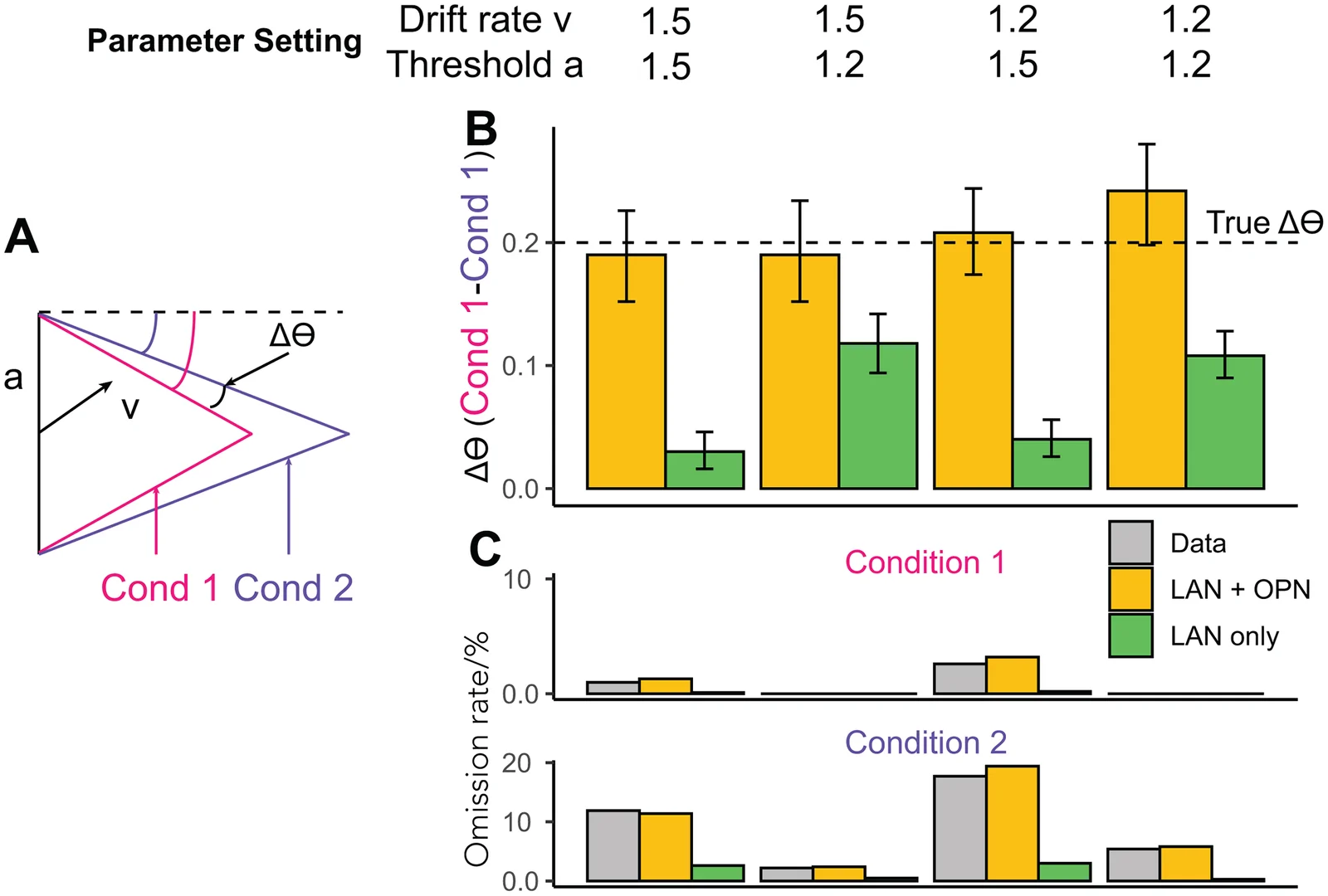
Estimation of change in boundary collapse in synthetic experiments. **(A)** Settings of synthetic experiments. Data is simulated from two conditions, with θ values of 0.9 and 0.7 respectively (true Δθ=0.2), for a variety of settings of drift rate (v) and initial threshold (a) parameters. **(B)** Recovery of collapse difference. While properly accounting for omissions correctly recovers Δθ, the parameter difference, ignoring omissions, severely underestimates it across conditions. Error bars indicate 95% confidence interval of posterior distribution. **(C)** Prediction of omission rate. Correspondingly, omission rates in posterior predictive distributions are wrongly calibrated if omissions are not appropriately accounted for during the inference stage. Models fit without omissions systematically predict much lower overall omission rates and smaller variance in omission rates.

In addition to the overestimation bias, we also sought to assess how taking omissions into account impacts a typical experimental setting in which one may test whether a model parameter varies by experimental condition. In this case, we simulate conditions in which θ differs across two experimental conditions (e.g., speed vs accuracy emphasis) but the analysis yields similar results with regard to the remaining model boundary parameters; Fig 4A). In a synthetic two-condition experiment, we found that the LAN-only approach underestimates the difference in θ between the two conditions (true Δθ=0.2; dashed line in Fig 4B). However, the LAN + OPN approach correctly recovers this difference between conditions (Fig 4B). This improved recovery of experimental condition on θ held across several combinations of the other model parameters drift rate and threshold that were tested (Fig 4B). Beyond parameter recovery, posterior predictive checks also confirmed that the LAN-only model fails to capture the true omission rate of each condition (Fig 4C) while including the OPN resolves this discrepancy.

### Identifying model parameters and lapse rate jointly

While the direct cause of omissions in our simulations so far is the response deadline, the underlying process leading to that omission could be multiply determined. We have assumed that omissions are due to not having hit the bound, but it is also possible that in empirical data, they reflect a lapse in attention and thus a different generative process in some proportion of trials. In empirical data analysis, researchers often include a lapse distribution (typically formulated as a uniform distribution of responses; [43]) to adjust the likelihood of outlier data points. Under a lapse distribution, omissions can result simply from sampling parts of the distribution with reaction times longer than the deadline. We have so far shown that, in the absence of explicit lapses, our model can effectively eliminate the omission-related bias in parameter recovery. However, when lapses are present, it is unclear whether our model can correctly attribute omissions to these lapses versus to the evidence accumulation process [31].

**Fig 5:**
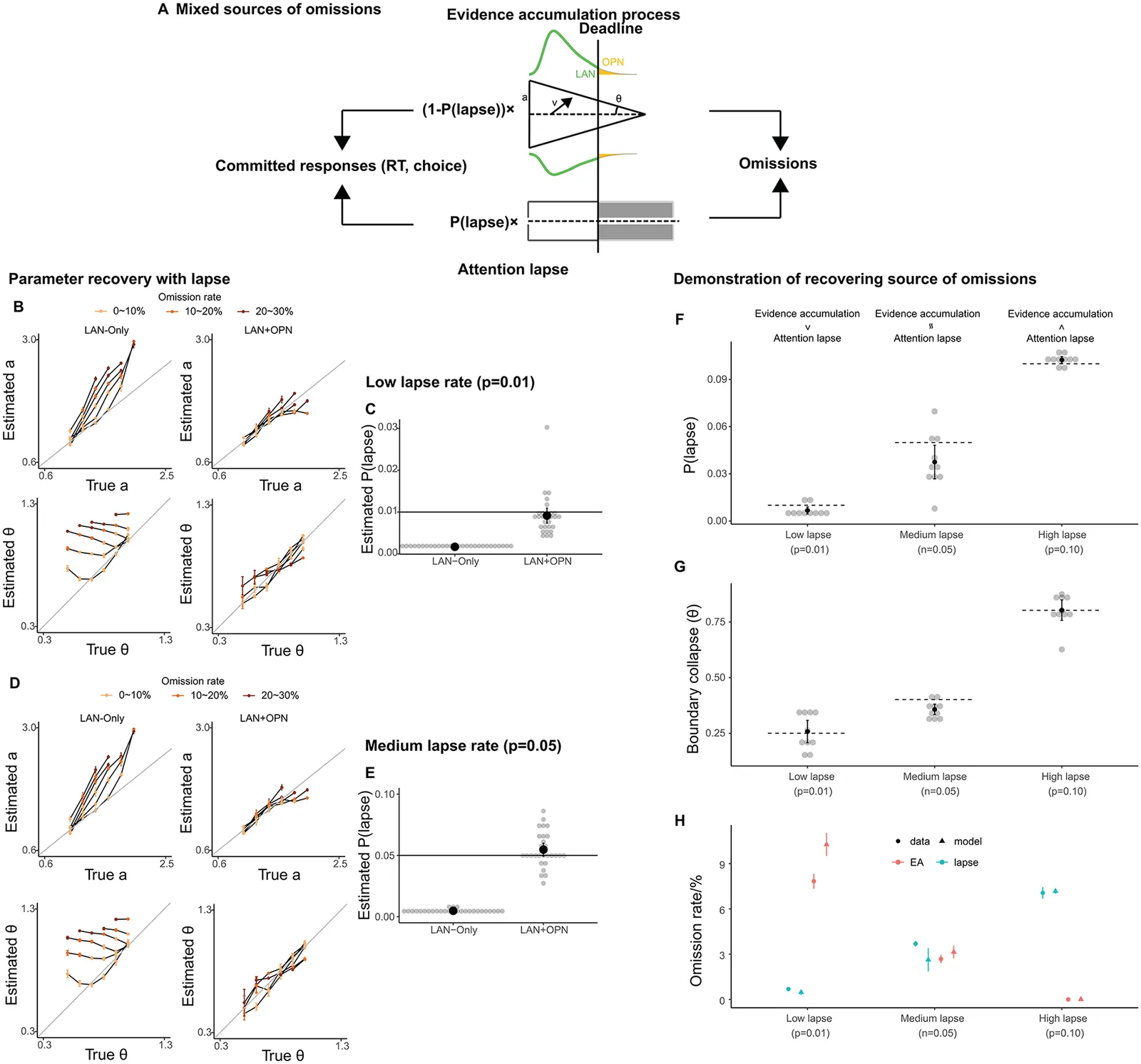
Parameter recovery when including lapse. **(A)** The model, whether considering omission or not, combines observed data (and omissions) generated from SSM and lapse process with a uniform distribution over rt and equal probability for choices. With a low lapse rate (p = 0.01; **B-C**), the LAN + OPN approach can properly recover both boundary parameters, while the LAN-only approach cannot **(B).** Illustrates the same phenomenon for the low lapse rate **(C).** This is confirmed with a higher lapse rate (p = 0.05; **D-E**). **Demonstration of recovering sources of omissions with LAN + OPN model (F-H)**. The model recovers the lapse probability **(F)** and boundary collapse **(G)**. The dominant source of predicted omissions aligns with the data **(H)**. Error bars indicate 95% confidence intervals across mean posteriors. Gray lines represent identity lines.

To address this question, we generated data jointly from SSM and a lapse distribution with a uniform probabilistic density function over a wide range of reaction time (Fig 5A). The proportion of data from the lapse distribution is indexed as $p_{lapse}$. For both the LAN-only method and the LAN + OPN approach, we characterized the distribution of observed data (and omissions for LAN + OPN) as a mixture of SSM and the lapse distribution, which allows us to estimate both parameters of the SSM and $p_{lapse}$. We tested the performance of parameter recovery with low (0.01) and high (0.05) values of $p_{lapse}$. Consistent with our previous results, our LAN + OPN approach properly recovered the parameters (a,θ) for both levels of $p_{lapse}$, while, as expected, a model without the inclusion of omission cannot recover the parameters (Fig 5B-5D). Importantly, we found that the LAN + OPN method not only recovered the parameters from SSM, but also provides accurate inference of the ratio of the lapse distribution, while a LAN-only approach significantly underestimated $p_{lapse}$, since a large proportion of the lapse distribution is represented as omissions and ignored (Fig 5C-5E). To confirm that the failure of lapse rate recovery is not due to specifics around our choice of lapse distribution, we implemented additional simulations when no omission exists. We found that our LAN-only model can then recover the lapse rate when omission is absent. As we apply more stringent deadlines, the lapse rate is underestimated in the model not considering omissions (see Fig A2 in S1 File).

To demonstrate that we can recover the sources of omissions, we ran additional simulations when omissions are 1) mostly from the evidence accumulation (EA) process; 2) equally from both EA process and attention lapse; and 3) mostly from attention lapse. We confirmed that, across three settings, our model can correctly recover the lapse rate (F) and collapsing boundary (G). Moreover, the model can predict the relative dominance between the two sources of omissions that aligns with the data (H).

### Extension to DDM with nonlinearly collapsing boundary

**Fig 6:**
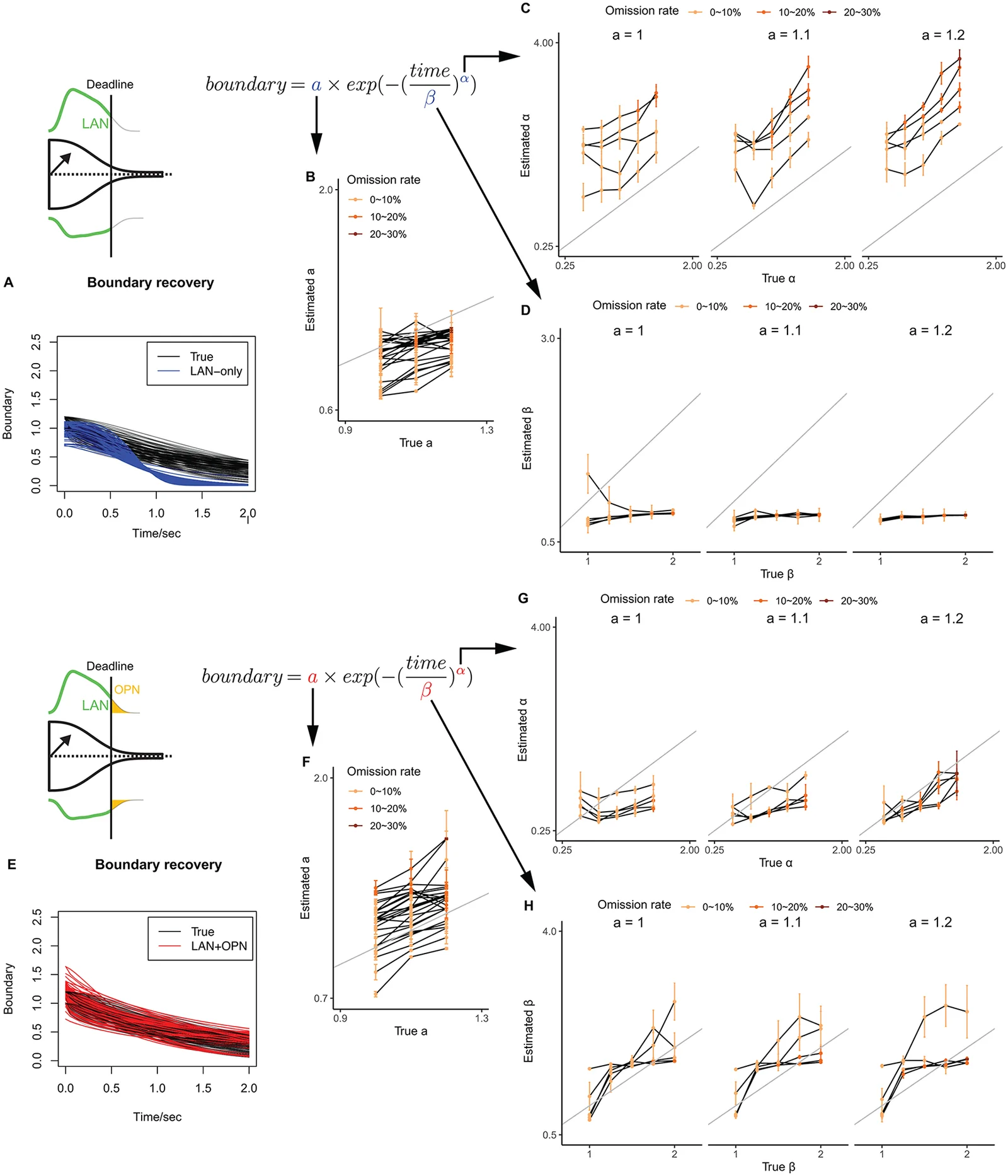
Parameter recovery performance for DDM model with a nonlinearly collapsing boundary. The boundary shape is characterized as a Weibull cumulative distribution function with initial threshold a, shape parameter α, and scale parameter β. Without considering omissions, a LAN-only approach failed to properly recover the boundary, with estimated boundaries collapsing down to nearly 0 much earlier than the true boundary **(A)**. Correspondingly, the model tends to underestimate initial threshold a **(B)**, overestimate shape parameter α **(C)**, and fail to capture most of the variance in scale parameter β **(D)**. The LAN + OPN approach recovers the true boundary much better **(E)**, with the recovery of all three boundary parameters improved **(F-H)**. Error bars indicate 95% confidence intervals of the mean posterior. Gray lines represent identity lines.

We have considered our approach for DDMs with either a constant or a linearly collapsing boundary. Past research suggests that the urgency process may not be constant over time, such that the boundary collapses nonlinearly. A typical formulation of this type of boundary shape is based on a Weibull cumulative distribution function (Fig 6; [16]). Since a nonlinearly collapsing boundary may better capture the dynamics of the urgency process, it is possible that such a model may be less vulnerable to omissions. We therefore sought to extend our approach to DDMs with a Weibull boundary, and test to what extent our model can improve parameter recovery under these conditions.

As suggested in past efforts of parameter recovery, it is difficult to recover the parameters of the Weibull boundary [38,46] unless the task includes multiple experimental conditions to help constrain parameter estimates [11], but the recovery of the overall boundary shape works much better. We first looked at the recovery of the Weibull boundary with the LAN-only approach (Fig 6A). Similar to the results of ANGLE model, the LAN-Only estimated boundary model collapses more steeply than true parameters in the generative process and with less variability at later time points, because the model does not consider the likelihood of omissions and is biased by observed RTs. When looking at the individual boundary parameters, the LAN-only model underestimated the initial boundary *a* (Fig 6B), overestimated the shape parameter α (Fig 6C), and underestimated the scale parameter β (Fig 6D). More importantly, regardless of the true scale parameter, the LAN-only approach always generated a similar qualitative estimation of the overall boundary shape (Fig 6D). This is consistent with the fact that the LAN-only model excludes variability in responding after the deadline (i.e., the omissions).

In contrast, with the LAN + OPN approach, the boundary shape was better recovered (Fig 6E). While the true parameters were still not perfectly recovered, which is a known identifiability limitation for this model when only a single experimental condition exists in the data [38], the LAN + OPN approach considerably improved parameter estimation bias relative to the LAN-Only approach. More specifically, LAN + OPN model reduced the bias in the initial boundary and shape parameter (Fig 6E-6F) and better captured variability in the scale parameter (Fig 6G). Comparing Figs 6A and 6E, we observe that, when omissions are present, recovered boundaries are systematically biased toward a highly non-linear shape in LAN-only model, even when underlying boundaries are much less non-linear. In contrast, with the LAN + OPN model the overall trend of boundary collapse is better recovered.

### Enabling the use of OPNs for the Scientific Community: HSSM

**Fig 7:**
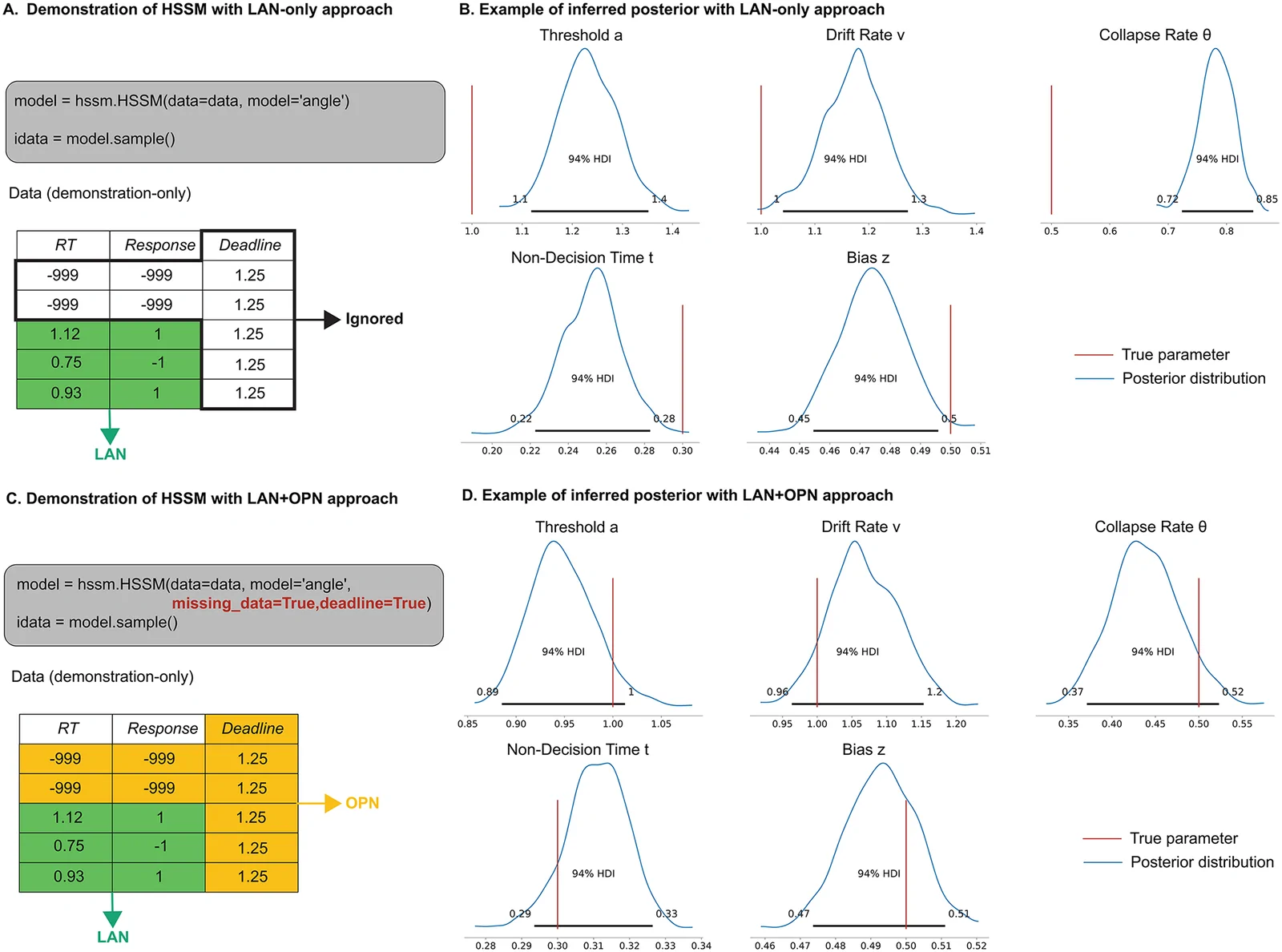
Demonstration of LAN + OPN approach in HSSM package (with ANGLE model as an example). **A)** Without specifying the existence of a deadline and correspondingly missing data, the default setting in HSSM will only calculate the likelihood of the observed data. **B)** In the example, the inferred posterior distribution deviates strongly from true parameter settings used for the corresponding synthetic dataset. **C)** Inclusion of OPN in HSSM can be specified by setting missing_data and deadline as True. The model will then incorporate the likelihood of both observed data and omissions. **D)** By doing so, the inferred posterior distribution is much improved and covers the true parameter, mirroring the core numerical results in this paper. The black bars in **(B)** and **(D)** indicate the 94% Highest Density Intervals (HDI) of the marginal posteriors for each of the inferred model parameters.

While we have thus far demonstrated the utility of OPNs for reducing bias in parameter estimation in custom code base, we also wanted to make the tool more readily usable for arbitrary modeling studies. We thus refined the implementation of the LAN + OPN approach for deployment in the **Hierarchical Sequential Sampling Model (HSSM)** python toolbox which enables hierarchical Bayesian estimation for a wide class of cognitive process models [47]. While the default setting in HSSM (Fig 7A) is to only include observed data in the inference and thus omissions (as well as the deadline information) will be ignored, we modified the toolbox to allow it to incorporate omissions via LAN + OPN. As a simple proof of concept, we reproduce the findings reported above: when omissions exist in the data, the default setting can lead to bias in the posterior distribution (Fig 7B). HSSM can incorporate the OPN with a simple configuration change (Fig 7C). Compared to the default settings, accounting for omission with OPN can improve the posterior distribution with better alignment with the true parameters (Fig 7D). This proof of concept using the ANGLE model can be easily extended in HSSM, where users can add additional LANs and corresponding OPNs for any arbitrary SSM.

## Discussion

When implementing cognitive models, it is common to apply heuristics to simplify the computational procedure. In the context of choice and RT modeling with deadlines, one such heuristic is to leave omissions out of the dataset when estimating computational model parameters. It is often assumed that this analysis choice is benign and without loss of generalization, especially when the overall proportion of omissions is relatively small. This assumption enables experimental scientists to incorporate response deadlines in their experimental paradigms and exclude omitted responses without raising concerns about the impact of these exclusions on inference. While this heuristic simplifies the computations to allow the use of standard toolboxes, its potential for contaminating model parameters has been overlooked and/or underestimated. Importantly, the lack of investigation into the consequences of this heuristic, especially for more complex variants of the DDM (SSMs), as the ANGLE and WEIBULL models, also results from the dearth of methods for properly (and computationally tractably) accounting for omissions to begin with [48].

Enabled by modern computational tools [4,38], in this paper, we sought to quantitatively examine the effects of this common heuristic of omitting omissions. We found that parameter recovery was severely impacted when ignoring omissions at inference, even when omissions are relatively rare (5% of trials or fewer). As we show via a synthetic experiment, this can result in highly misleading conclusions when comparing parameter values across separate experimental conditions, in search of mechanistic explanations of the effects of experimental manipulations. We demonstrated that this consequence remains even when accounting for attentional lapses and more complex boundary shapes (e.g., WEIBULL model). On a broader scale, we think that this investigation points to the value of continued re-examination of collective methodological choices. Taking advantage of the continuous development of novel inference tools [33], such revised efforts become progressively more feasible at the frontier of the computational sciences.

The lapse process utilized in our current framework structurally mirrors the standard uniform contamination process formalized by [43]. However, we acknowledge that this is not the only way to model lapses. Recent literature also proposed more dynamic, mechanistic accounts of off-task states; for instance, work in [49] explores lapses through the lens of mind wandering, modeling structural shifts in continuous attention rather than static noise (see [50] for similar models in animal studies). While our current model leverages the standard contamination approach for its parsimony, future extensions of this work would benefit from a more thorough integration of these diverse theoretical perspectives, exploring how dynamic attentional models might further refine our understanding of omissions in evidence accumulation frameworks.

The scarcity of computational investigations into this effect can be attributed to the analytical challenges it has previously posed. The proper integration of deadlines into likelihood computations requires substantial effort compared to the standard omission-ignorant workflow supported by canonical tools [35,51–53]. These challenges prevented previous work from explicitly modeling omissions, and examining the extent to which estimated parameters deviate from the ground-truth when these omissions are excluded from the modeled data. The framework of simulation-based inference [33] -- particularly the learning of likelihood functions directly from simulation [36,38,54] -- offers a promising general-purpose approach. This approach can be applied productively to not only the influence of omission in our investigation, but also a much broader class of cognitive computational models and experimental designs.

Extending from LANs, our approach provides an accessible tool to help researchers properly incorporate omissions into their decision making models. We have demonstrated that this approach can effectively reduce the influence of omission on parameter estimation, across different boundary shapes and robust to the existence of lapse distribution. We hope our work makes the decision about how to work with omissions in data easier. Similar to LANs, this new LAN + OPN approach is designed to suggest a workflow that exposes the moving pieces in a computational modeling endeavor explicitly, while at the same time streamlining the otherwise analytically tedious aspects that adjustments of likelihood computations entail, through strong reliance on simulation. This approach fundamentally enabled the present investigation and is available to the wider community via the public HSSM python package [47]. Taking omission as an example, our work elucidates why it is not optional, but necessary, to use these modern tools to help diffuse computational best practices in the quest to avoid misleading conclusions from scientific work based on computational modeling.

## Supporting information

S1 File Supporting Information. (DOCX)
