# Supplementary material for: The perils of omitting omissions when modeling evidence accumulation
Source: PLoS Comput Biol. 2026 Aug 21;22(8):e1014667. doi: 10.1371/journal.pcbi.1014667 (PMC13521452; doi:10.1371/journal.pcbi.1014667)
Supplement: Supplementary file 1 [file pcbi.1014667.s002.pdf]

Comments to the Authors:
Please note here if the review is uploaded as an attachment.

Dear editor and reviewers,

Thank you for the comments and feedback. We really appreciate the time and effort in this process.

We have revised the manuscript based on the comments. Below you will find our responses to each of them in blue ink color. We hope our responses and revisions can address your concerns.

Reviewer #1: In The Perils of Omitting Omissions when Modeling Evidence Accumulation, the authors study the effect of ignoring missed trials on parameter estimation in diffusion decision models of decision making behavior. Using simulations, they demonstrate that ignoring omissions can lead to biased parameter estimates. They show that unbiased estimates can be obtained by incorporating the probability of response omission into the likelihood function. To enhance computational efficiency, they propose the use of likelihood approximation networks and omission probability networks.

Overall, the paper is well-written and, for the most part, easy to follow. The authors' argument that omissions should be included in the likelihood function is theoretically sound and convincing. However, I am less convinced about the practical relevance.

Specifically, my primary concern with the paper is the likelihood that current practices of omitting omissions significantly influence the literature. The authors do not provide empirical data on the actual omission rates reported in the literature. Their simulations show that higher omission rates lead to more biased parameter estimates, but these simulations use parameter settings that result in omission rates of 10% to 30%. Such rates are unusually high. For this review, I briefly examined some open-access datasets* that I am familiar with and found omission rates between 1% and 2%. It also makes sense that these rates are so low, since participants are normally penalized for missing responses in one way or another, and they adjust their behavior accordingly to ensure they respond in time. Even in paradigms that are explicitly designed to test omission rates, the reported omission rates are in the range of 2% to 6% (e.g., Damaso et al., 2022, 10.3758/s13421-021-01265-z). Additionally, there already are methods to account for omission rates, and these have been applied in cases where omissions were hypothesized to potentially influence the results (e.g., Howard et al., 2020 10.3758/s13423-020-01741-8; Evans et al., 2018, 10.1111/cogs.12627).

This need not be the case in all studies, and perhaps my selection from the literature is just biased. But are there then studies where the omission rates are so high? Perhaps in clinical studies?

Response:

We thank the reviewer for pointing out the lack of literature support for our argument in this paper. We have conducted literature searches and collected representative papers with omission rates greater than 2%. We have included this table in the Supporting Information.

Table S1

Paper	Deadline/ms	Mean OR/%	Range of OR across experiment conditions/%	Range of OR across subjects/%
Pleskac, 2018	Study 1: 850<br>Study 2: 630	Study1: 3<br>Study2: 10	Study1: 1~5<br>Study2: 5~15	Study1: 0~10<br>Study2: 1~38
Grahek, 2022	750	7	5~10	0~30
Evans, 2020	Experiment 2: 1300	2.8	1~4.6	0~8<br>(Note: subjects with omission rate>10% were excluded)
Katsimpokis, 2020	Experiment 3: 683, 1350 or no deadline	For all conditions with deadline: 5.8	683 ms: 8.8~10.8<br>1.35s: 0.8~2.6	683 ms: 2~28<br>1.35 s: 0~6.5
Teoh, 2020	Study1:<br>1.5 s or 10 s	2.5	1.5 s: 4.7<br>10 s: 0.3	1.5 s: 0~17<br>10 s: 0~2.5

More importantly, while it is true that overall omission rate is not very high (e.g., overall omission rate < 5%) in some studies, the omission rate is not uniformly distributed between and within subjects, so that in some conditions and some subjects, the omission rate can be as high as >10%. The intuition to investigate the influence of omission in a wide range of omission rate is to make this paper more inclusive about the potential influence on between- and within-subject effects. It is worth noting that even for these existing datasets, some did not record the trials with omissions so we have to infer the omission rate based on trial numbers (for example, Katsimpokis et al., 2020). It is possible that this reflects a common practice that could make the omission rate generally underestimated.

Meanwhile, as we have included in this revision in response to Reviewer #2, we showed that for the standard DDM, the bias in estimation of threshold and drift rate is present even when the omission rate is as low as 5%. We also investigated the influence of omission on model recovery, and found that even at low omission rate (<5%), model recovery can bias toward a DDM with collapsing boundary even though the generative process is with constant boundary.

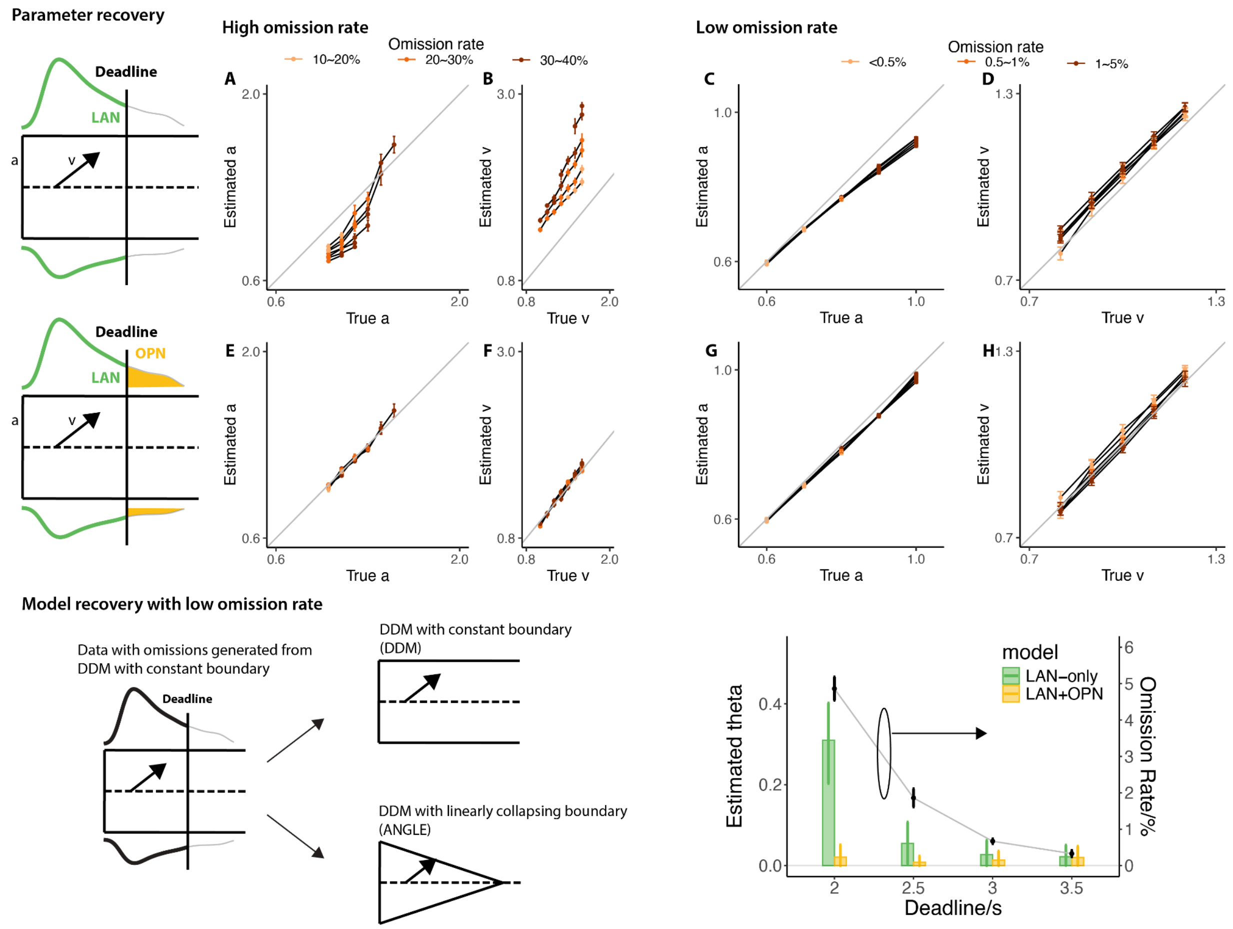

Figure 2. Parameter recovery performance for DDM model with constant threshold, when either ignoring omissions (e.g., only considering likelihood of responses before deadline; LAN-only model; A-D) or including them via the omission probability network as explained in Figure 1 (e.g., including integral of post-deadline likelihood; LAN+OPN model; E-H). When omission exists, the LAN-only approach underestimates response threshold a (A) and overestimates drift rate v (B). This bias emerges even when omission rate is lower than 5% but higher than 1% (C, D). With the LAN+OPN approach, both threshold a (E, G) and drift rate v (F, H) are properly recovered. Model recovery for DDM model with constant threshold (I). When omissions are ignored (LAN-only model), the posterior distribution of boundary collapse deviates from ground truth (zero) even when omission rates is less than 5%, while LAN+OPN model can properly recover the constant boundary. Error bars refer to 95% confidence intervals of mean posteriors across multiple synthetic data sets from the same parameter configuration. Gray lines represent identity lines.

Additionally, in their initial simulations, the authors assume that omissions are solely the result of the accumulated evidence not reaching the threshold yet—referred to as task omissions in Damaso et al.'s terminology. However, as the authors acknowledge, this may not be the only or even the primary source of omissions. Other possibilities include attentional lapses and/or an intrinsic lack of evidence accumulation. While the authors address the attentional lapse hypothesis, I found the setup of this simulation difficult to understand and struggled to interpret the results. Specifically, why is the lapse rate

not recovered at all with LAN-only models? Do the LAN models recover the lapse rate in the absence of omissions? The LAN-only panels in Figures B and D appear identical; is this correct?

Response:

The reviewer raised an important concern about our recovery of lapse probability. We have included more details about the simulation steps in the text:

In Methods we updated and include the reference about the contaminant process:

> While our primary analysis assumes that all choices are generated from a single SSM parameterization, empirically, it is also possible that some proportion of responses and omissions reflect attentional lapses. To test how our approach performs in this case, we followed the setup of contaminant process in [43] and modeled a lapse distribution process as a uniform distribution over a wide range of time T (5s in our analysis) and assuming that lapses generate random choices (in this case, p=0.5 for each choice option)

To confirm that the failure of lapse rate recovery is not due to how we set up the lapse distribution, we implemented additional simulations when no omission exists. We found that our LAN-only model can then recover the lapse rate when omission is absent. As we apply more stringent deadlines, the lapse rate is underestimated in the model not considering omissions.

Our interpretation is that, as the lapse distribution generates a relatively larger proportion of long-RT responses than the evidence accumulation process, ignoring omissions implies removing omissions mostly from the lapse process. We have included this simulation in the Supporting Information.

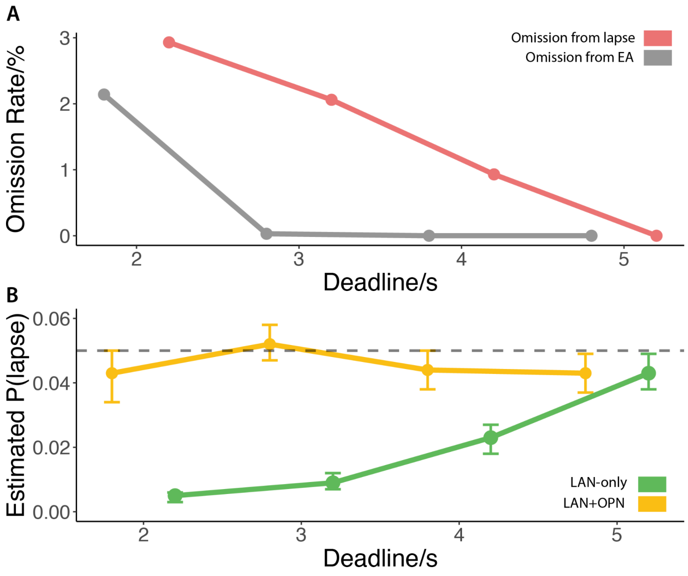

Figure S2. Recovery of lapse with and without the existence of omissions. The omissions from evidence accumulation (EA; for this demonstration we used ANGLE model) and lapse increases with more stringent deadline (A). Across different deadline and omission rates, LAN+OPN model can properly recover the lapse probability, while for LAN-only

model the lapse probability can only be recovered when no omission from lapse exists (B).

We thank the reviewer for the reminder concerning potential issues in the plot. We have checked data from panel B and D. Both are similar as the estimated lapse rates are very low with the LAN-only approach but they are not identical.

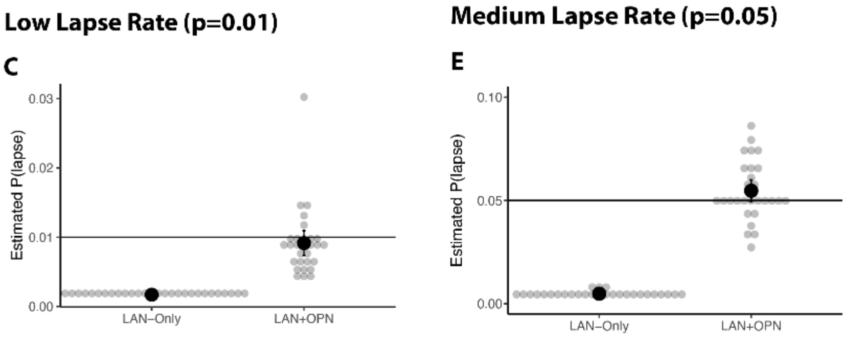

More importantly, I believe the setup of this simulation overlooks a critical question. Given an empirical dataset, we do not know the source of omissions—it could be missed deadlines or attentional lapses. The current simulation assumes that omissions are primarily due to missed deadlines (even the 'high' lapse rate condition has a probability of only 0.05, compared to up to 30% omissions due to missed deadlines) and then demonstrates that the parameters can be recovered. However, can the presented methods also be used to determine whether the omissions are due to missed deadlines or attentional lapses?

Response:

To demonstrate that we can recover both lapse rate and boundary collapse separately when the rate of omissions are comparable between these two cases, and thus to distinguish the source of omissions, we ran additional simulations when overall omission rate is less than 10% and:

1) When most omissions are from the decision process
2) When the rate of omission from two processes are similar
3) When most omissions are from the lapse process

We found/reconfirmed that, across three settings, our model can 1) correctly recover the lapse rate; 2) correctly recover the collapsing boundary and 3) can predict a similar ratio of the two kinds of processes. We hope that this can address the point about whether our approach can infer the proportion of omissions from different processes.

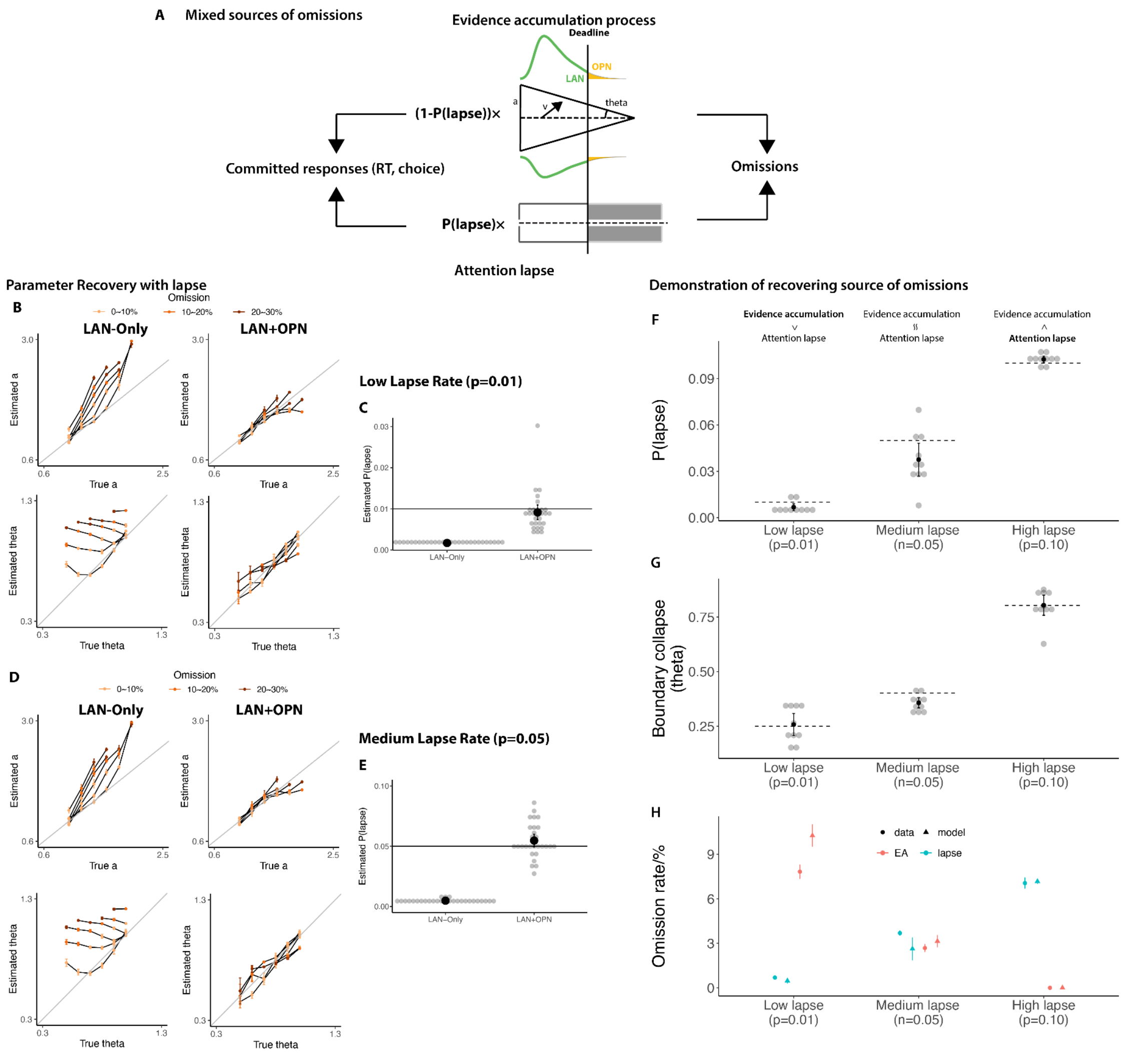

Figure 5. Parameter recovery when including lapse. (A) The model, whether considering omission or not, combines observed data (and omissions) generated from SSM and lapse process with a uniform distribution over rt and equal probability for choices. With a low lapse rate (p=0.01; B-C), the LAN+OPN approach can properly recover both boundary parameters, while the LAN-only approach cannot (B). Illustrates the same phenomenon for the low lapse rate (C). This is confirmed with a higher lapse rate (p=0.05; D-E). Demonstration of recovering sources of omissions with LAN+OPN model (F-H). The model recovers the lapse probability (F) and boundary collapse (G). The dominant source of predicted omissions align with the data (H). Error bars indicate 95% confidence intervals across mean posteriors. Gray lines represent identity lines.

Finally, I found the content of the paper highly focused on the DDM, while it is framed as a paper investigating SSMs more generally. How large is the influence of neglecting omissions in race models? Is there perhaps a stronger case to be made there?

Response:

Thank you for this excellent suggestion. We have conducted additional simulations using the race model to investigate the impact of omitting omissions. Our results demonstrate that, similar to the DDM, omitting omissions leads to biased parameter estimates in race models, particularly concerning drift rates and decision thresholds. These findings confirm that accounting for omissions is critical across multiple sequential sampling models. Specifically, our simulations show that omitting omissions biases parameter estimation by inflating both drift rates, while maintaining the relative difference between them. Given the consistency between the results with DDM and race model, we decided to put this into the Supporting Information.

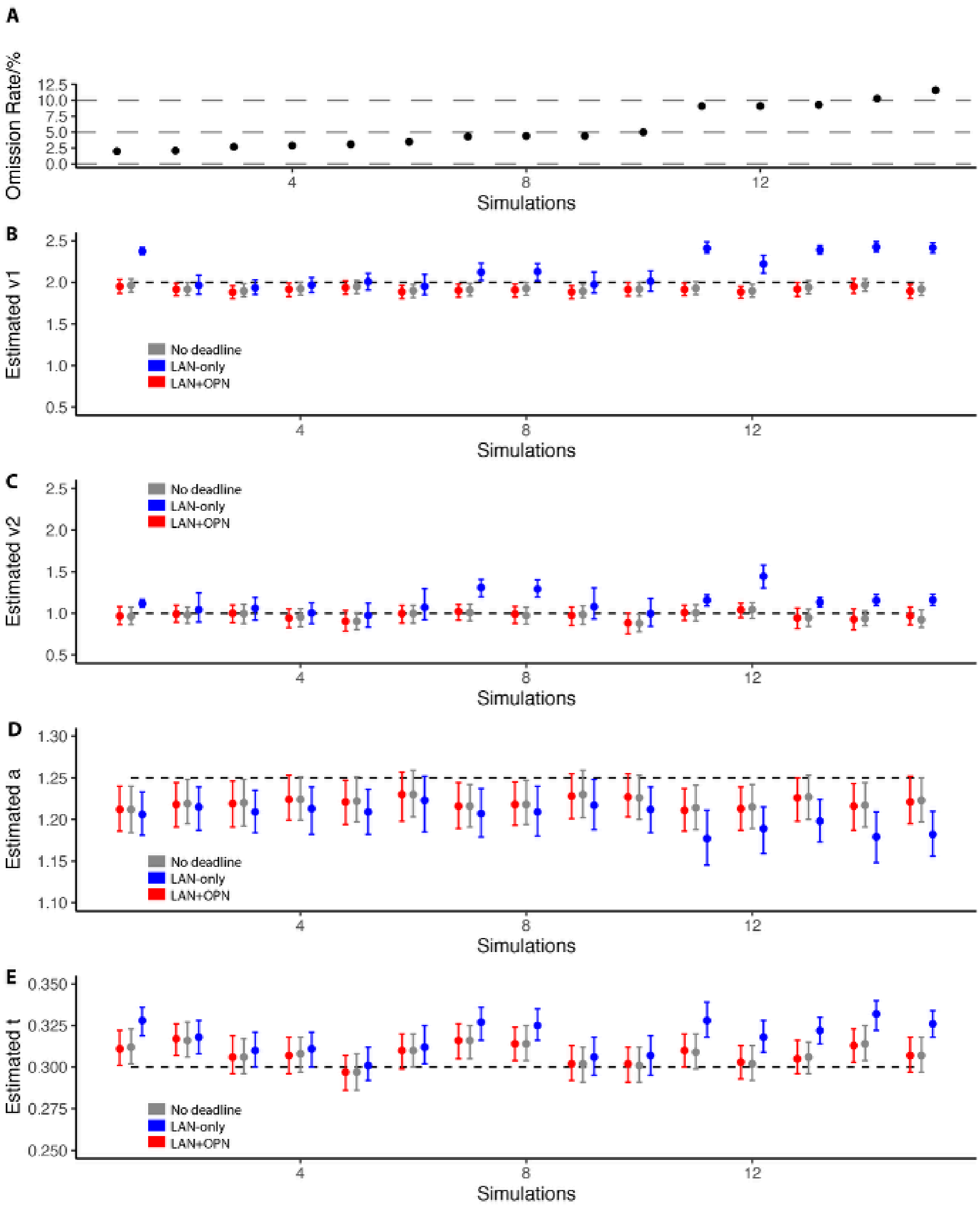

Figure S1. Parameter recovery of race model with/without including omissions. Across different levels of omission rate (A), LAN-only model overestimates drift rate for both accumulators and underestimate threshold when omission rate is between 5~10 %, compared to the recovered posterior when no omissions exist (e.g., no stringent

> deadline). Additionally, the model tends to overestimate non-decision time. With the LAN+OPN model, the parameter recovery aligns with the cases when no omission exists.

Minor points

p. 3, line 87: “recent extensions of SSM models”. This is really very minor but the work on urgency has been around for a while now, I wouldn’t call it ‘recent’ anymore.

We have updated the text to accurately reflect the literature.

p. 3, line 89-93: The (critical) study on optimality of collapsing bounds by Boehm et al. (2020) should be mentioned here: 10.3758/s13414-019-01806-4

We have included this paper into our references.

p. 5, line 143: The parametrisation of z and a should be clarified. If the bounds are at [-a, a], a start point of z=0 is unbiased. If the bounds are at [0, a], a start point of z=0.5*a is unbiased.

We thank the reviewer for pointing out this inconsistency in the parameterization of the model. The boundary is parameterized as [0,2a] while the starting point of z=a (e.g., 0.5 * 2 * a) is unbiased.

p. 5: Are between-trial variabilities in drift rates, start points, and non-decision times included in the simulations?

We did not include between-trial variabilities in the simulations. We have added a note in the methods part about this.

> For the sake of simplicity, we only consider models without inter-trial variability of drift rate, starting point bias and non-decision time. We expect that our findings can be generalized to those models.

It would be an important future step to test how omission influences models including these parameters, but to establish the necessity of including omission, we believe our current evidence is sufficient.

p. 10, line 245: “Larger than accurate bias parameters” – Maybe I am missing something, but Figure 2B doesn’t include bias (start point) parameters?

We thank the reviewer for pointing out this. This is a typo and we have corrected it.

p. 18, line 362: Evans et al., (2019) should be included in the discussion of the recoverability of time-varying parameters such as collapsing bounds: 10.3758/s13428-019-01218-0

We really appreciate this suggestion and we have included this literature in the discussion.

p. 18–20: I had difficulty understanding the rationale for including the non-linearly collapsing boundary case. As the authors acknowledge, parameter recovery for such models is very poor, even in the best-case scenario with no omissions. From what I understand, the authors suggest that, despite the inability to recover the parameters accurately, it might still be possible to recover the shape of the boundary. However, Figure 6E appears to show bounds that are very close to linear, although it is challenging to interpret the panel clearly.

The reviewer raised an important concern about the role of non-linearly collapsing boundary parts. As the reviewer mentioned, we are more interested in the recovery of the shape. Comparing Figure 6A and

Figure 6E, we tried to highlight the difference between LAN-only and LAN+OPN. Parameter recovery is genuinely difficult for this (Weibull) model because trade-offs between boundary parameters exist-in other words many candidate parameter settings can produce similar looking boundaries. However, with a focus on shape, instead of specific parameters, we observe that the recovered boundary when omissions are present and we use the LAN-only model, we can get boundaries that are systematically biased toward a highly non-linear shape, even when the underlying boundary is much less non-linear. In contrast, with the LAN+OPN model we can better recover the overall trend of boundary collapse. We have made the emphasis on shape vs. parameters more clear in the text.

*datasets/papers:
- Wagenmakers et al. (2008; 10.1016/j.jml.2007.04.006), experiment 1: 2% excluded, experiment 2: 1.5% excluded (range 300 – 2500 ms);
- Miletić & van Maanen (2019; 10.1016/j.cogpsych.2019.01.002) exp 1: deadline 1.5 seconds, in total 77 missed responses, out of 18868 trials (across all subjects)
- Forstmann et al. (2008; 10.1523/JNEUROSCI.1465-08.2008): Behavioral session, 1.5 seconds stimulus, on average 2% of trials excluded. One subject had 18% omissions – when excluding that participant, the average omission rate was 1.1%. These exclusions include fast guesses (<0.15 s response) as well as missed deadlines.
- Damaso et al., (2022) use an explicit distraction paradigm intended to study omissions, and even then they find only 6.4% omissions in elderly subjects, and 2.2% in young subjects.

Reviewer #2: The current manuscript assesses the impact of ignoring omissions (i.e., non-responses) in typical experimental data. Specifically, the current manuscript assesses the impact of ignoring omissions on the parameter recovery of different evidence accumulation models, where the generating process produces data that would result in omissions (i.e., response times longer than the response deadline). The current manuscript finds that parameter recovery is greatly impaired when these omissions are ignored, but is near perfect (at least, for regular diffusion models) when these omissions are explicitly included in the model.

I have mixed feelings about the current manuscript. On the one hand, I think it's very interesting work, and the results make it clear that data with high omissions produce unreliable parameter estimates without the inclusion of omissions. On the other hand, I feel as though the contributions are somewhat overstated, and the simulations aren't as thorough (and/or are lacking important details) as they could be. Therefore, I think that the current manuscript will make a nice contribution to the literature, but requires some revision both in terms of the literature discussed and the scope of the simulations, which I detail below.

(1) The idea of omissions is something that, in my opinion, has had a decent amount of research put into it, and has been addressed in evidence accumulation models previously. However, the current manuscript ignores a lot of the existing literature, indirectly overstating its contribution. For example, I find it crazy that the seminal paper on omissions in response time data, which the current manuscript indirectly utilises, isn't even cited (Ulrich & Miller, 1994; Effects of truncation on reaction time analysis). Personally, I've modelled omissions in evidence accumulation models in several situations, and the idea of modelling omissions forms the basis for my double responding models (e.g., Evans et al., 2020; Double responding: A new constraint for models of speeded decision making). Furthermore, the lapse process seems to just be the standard Ratcliff contamination process, which I think should be more prominently noted (and that this isn't the only way to think about "lapses", e.g., see Guy Hawkins' work on mind wandering). I'm only

naming the first things that come into my head, but there's a lot more usage of omissions modelling than the current manuscript currently indicates, and I think that the authors need to be much more thorough in their discussion of the literature in a revision.

Response:

We thank the reviewer for pointing out the past work we have missed in the paper and we fully agree that the issue of omission has been raised before. We have included more detailed discussion about past literature in our introduction and limitation/future work in the discussion. We would like to clarify that the aim of this paper is not about raising a new issue about omission, but to establish the caveats of the most common methods in evidence accumulation modeling when dealing with omissions, which has not been systematically investigated in past literature. We hope that, with the inclusion of the suggested past work, we are making this point clearer.

In the introduction:

> It is crucial to recognize that the profound implications of omitted responses and data truncation on reaction time distributions have been raised in [29].

and

> Recently, evidence accumulation models have sought to incorporate omissions [30,31]. While it can be easy to derive omission probability from the cumulative distribution function of responses for some cases that permit analytical calculations [31], it becomes computationally difficult in many cases, even for simple variations of canonical cognitive process models. Such "intractable" models, however, are becoming more popular as researchers are interested in accounting for neural nonlinearities and other time-varying processes during decision making [11,16]. Numerical and simulation-based methods have been implemented to accommodate omissions [32,33]. However, the availability of these methods does not equate to a low practical burden. The numerical approach [32] requires extensive computation to obtain probability density function. Simulation-based inference improves the calculation [30], but does not handle dataset with hierarchical design naturally. Existing packages (for example, HDDM, SBI, EMC2; [34-36]) for evidence accumulation models generally lack native support for non-responses. Therefore, researchers might have to handle simulations, mixture likelihoods, and hierarchical structures to account for omissions. All of these could increase the burden of model usage, frequently biasing researchers toward the heuristic approach of ignoring omissions entirely.

In the discussion:

> The lapse process utilized in our current framework structurally mirrors the standard uniform contamination process formalized by [43]. However, we acknowledge that this is not the only way to model lapse. Recent literature also proposed more dynamic, mechanistic accounts of off-task states; for instance, work in [49] explores lapses through the lens of mind wandering, modeling structural shifts in continuous attention rather than static noise (see [50] for similar models in animal studies). While our current model leverages the standard contamination approach for its parsimony, future extensions of this work would benefit from a more thorough integration of these diverse theoretical perspectives, exploring how dynamic attentional models might further refine our understanding of omissions in evidence accumulation frameworks.

(2) I think that the description of the omissions process is made much more complex than it needs to be, and the computational burden of implementing this using standard approaches is overstated. In truth, the probability of an omission occurring is just the survivor function of the accumulation process (i.e., 1 – CDF_1 – CDF_2 … – CDF_n), and this doesn't add too great of a computational burden for analytic models (e.g., see Evans et al., 2018; Modeling the covariance structure of complex datasets using cognitive models: An application to individual differences and the heritability of cognitive ability; or see Howard et al., 2020; How is multi-tasking different from increased difficulty?) or for simulation-based methods (e.g., see Evans et al., 2020; Double responding: A new constraint for models of speeded decision making). I agree that it adds a problematic computational burden for numeric methods for calculating the likelihood (i.e., if you already require integral estimation for getting the PDF, then getting the CDF is quite problematic), but I think these situations are becoming more rare (e.g., I used a numeric method for collapsing thresholds models with deadline data in Evans et al., 2020; The role of passing time in decision-making; which made modelling omissions too computationally taxing, but I think most people would use simulation-based methods these days, as they're faster). I still think the current framework is useful, but I disagree with the argument that the omissions method cannot be (or is too computationally taxing to) implement in existing frameworks.

Response: The reviewer raises important points about the existing approaches of omission probability calculations, and we appreciate the specific references and clarifications. We have included these literatures in the introduction and highlighted existing approaches.

In the introduction, we updated:

> Recently, evidence accumulation models have sought to incorporate omissions [30,31]. While it can be easy to derive omission probability from the cumulative distribution function of responses for some cases that permit analytical calculations [31], it becomes computationally difficult in many cases, even for simple variations of canonical cognitive process models. Such "intractable" models, however, are becoming more popular as researchers are interested in accounting for neural nonlinearities and other time-varying processes during decision making [11,16]. Numerical and simulation-based methods have been implemented to accommodate omissions [32,33]. However, the availability of these methods does not equate to a low practical burden. The numerical approach [32] requires extensive computation to obtain probability density function. Simulation-based inference improves the calculation [30], but does not handle dataset with hierarchical design naturally. Existing packages (for example, HDDM, SBI, EMC2; [34-36]) for evidence accumulation models generally lack native support for non-responses. Therefore, researchers might have to handle simulations, mixture likelihoods, and hierarchical structures to account for omissions. All of these could increase the burden of model usage, frequently biasing researchers toward the heuristic approach of ignoring omissions entirely.

(3) I disagree with the general idea that response deadlines are commonly used to incentivize speedy responding. In general, when it comes to experimental psychology, I think the most common method to incentivize speedy responding are the classic SAT emphasis instructions (i.e., just telling people to focus on speed or accuracy), and deadlines are usually avoided for the censoring/omissions reasons discussed in Ulrich & Miller (1994) and in the current manuscript (unless deadlines are specifically of interest). I know the use of deadlines are more common in other areas, though (e.g., I think this is the case in

decision neuroscience?), so perhaps it would be good to be more specific in discussing what areas typically use response deadlines (and maybe including more citations to usages).

Response:

The reviewer raised an important missing part in our paper regarding the specific scenarios when response deadline is used. We agree that instructions/cues are commonly used to induce speed-accuracy trade-off. Meanwhile, we have noticed that past work has shown that deadline-based speed-accuracy manipulation might introduce different mechanisms compared to the instruction-based approaches, as discussed in Katsimpokis et al, 2020 ("Not all speed-accuracy trade-off manipulations have the same psychological effect").

As the reviewer suggested, we have found a few representative literature examples with deadline manipulation in different research questions. We have included these references into the paper.

1. Animal research about speed-accuracy tradeoff (where instruction-based manipulation is not feasible):
   Reppert, T. R., Heitz, R. P., & Schall, J. D. (2023). Neural mechanisms for executive control of speed-accuracy trade-off. Cell reports, 42(11).
2. Recent work that explicitly used deadline to manipulate time pressure:
   Stone, C., Mattingley, J. B., & Rangelov, D. (2025). Neural mechanisms of metacognitive improvement under speed pressure. Communications Biology, 8(1), 223.
3. Cognitive control in speeded decision making:
   Vassena, E., Deraeve, J., & Alexander, W. H. (2020). Surprise, value and control in anterior cingulate cortex during speeded decision-making. Nature human behaviour, 4(4), 412-422.

We have updated the relevant part in the introduction to clarify the scope of research questions with usage of response deadlines.

In introduction:

> In practice, while deadlines have been used to induce speedy decision-making and manipulate time pressure, especially when studying the underlying neural dynamics [18-22], it is often inevitable to have trials for which no response is given (e.g., omissions; see Table S1 in Supporting information for examples; [18,22-25]).

Moreover, empirically we note that multiple ongoing collaborations of the authors deal with deadlines scenarios and, before our consultation on data analysis, had defaulted to omitting omissions. Through the development of the HSSM toolbox, the authors in fact witnessed multiple user questions suggesting the same default behavior concerning omitting omissions and access to an uploaded OPN is popularly requested by academics. This lived experience corroborated our cultural assumption that the practice to omit omission is widespread, and prompted us to consider the effects on parameter estimation in detail.

(4) Building on the last point, while some type of censoring is typically used in response time tasks, either through deadlines or excluding "long" responses that are thought to be contaminants (e.g., the participant lost attention), the censoring is usually much later in time (e.g., 5s), and typically excludes relatively fewer trials than is the case in these simulations. While the Supporting Information do provide recoveries using a slightly different deadlines (1s or 1.5s, vs the 1.25s in the main text), this is only for the linear collapsing threshold model. Importantly, I would be much more interested to see how the standard diffusion model (i.e., the most used model for parameter estimation in real data) performs with much fewer omissions

(and preferably, a much longer response deadline), as I think this is crucial for claims like “We found that parameter recovery was severely impacted when ignoring omissions at inference, even when omissions are relatively rare (5% of trials or fewer).”

Response:

The reviewer pointed out an important mismatch between the real cases and our simulation studies. As we have responded to Reviewer #1, there are different scenarios where the setting of response deadline induces relatively higher omission rate. We have included this table in the Supporting Information.

Table S1

Paper	Deadline/ms	Mean OR/%	Range of OR between conditions/%	Range of OR between subjects/%
Pleskac, 2018	Study 1: 850<br>Study 2: 630	Study1: 3<br>Study2: 10	Study1: 1~5<br>Study2: 5~15	Study1: 0~10<br>Study2: 1~38
Grahek, 2022	750	7	5~10	0~30
Evans, 2020	Experiment 2: 1300	2.8	1~4.6	0~8<br>(Note: subjects with omission rate>10% were excluded)
Katsimpokis, 2020	Experiment 3: 683, 1350 or no deadline	For all conditions with deadline: 5.8	683 ms: 8.8~10.8<br>1.35s: 0.8~2.6	683 ms: 2~28<br>1.35 s: 0~6.5
Teoh, 2020	Study1:<br>1.5 s or 10 s	2.5	1.5 s: 4.7<br>10 s: 0.3	1.5 s: 0~17<br>10 s: 0~2.5

Additionally, we have run simulations with omission rate < 5% for standard DDM. As included in the paper, we have found that the LAN-only model systematically underestimates the threshold at least when overall omission rate is greater than 1%, while overestimating the drift rate. With the inclusion of omission in the model, the overall parameter recovery performance is improved.

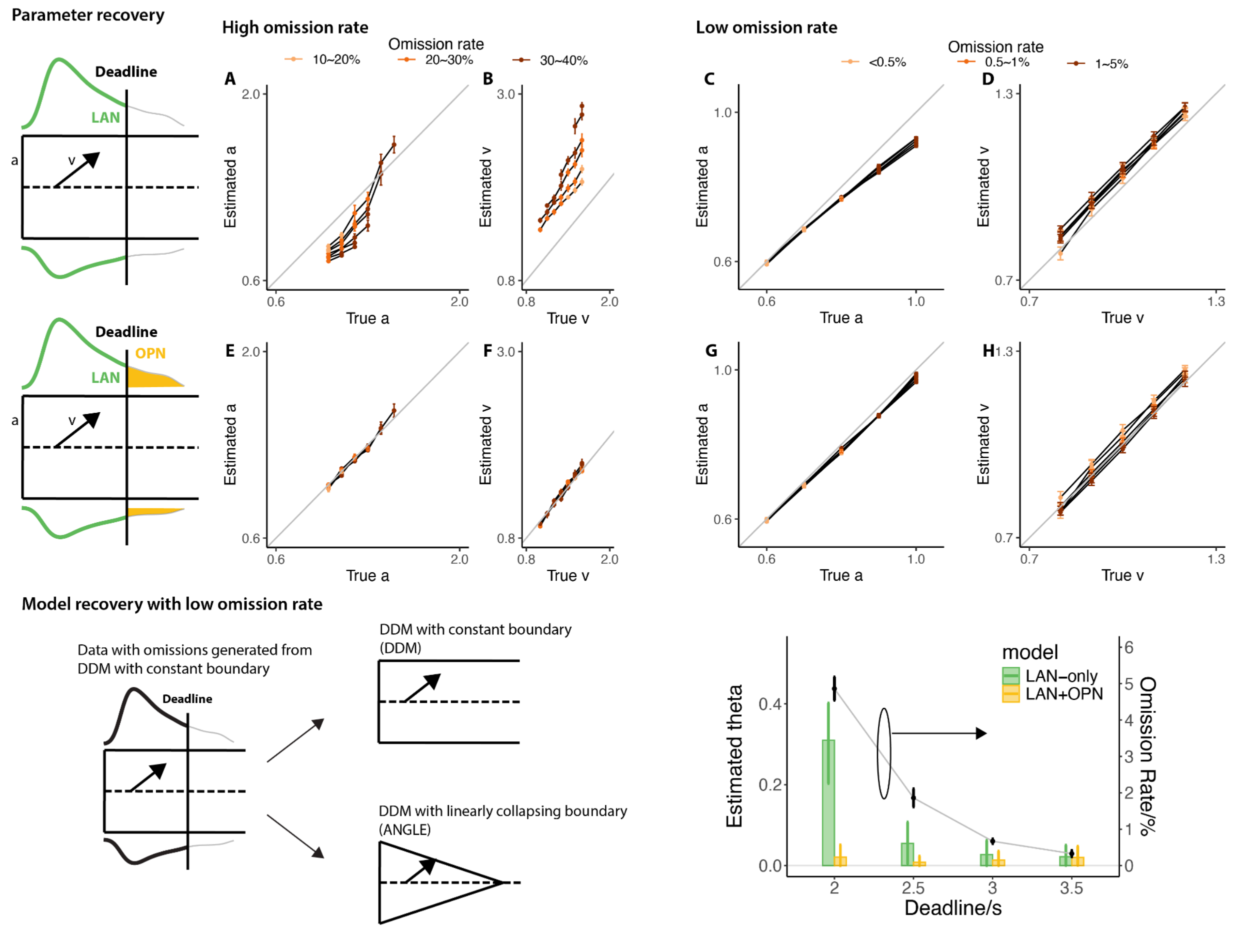

Figure 2. Parameter recovery performance for DDM model with constant threshold, when either ignoring omissions (e.g., only considering likelihood of responses before deadline; LAN-only model; A-D) or including them via the omission probability network as explained in Figure 1 (e.g., including integral of post-deadline likelihood; LAN+OPN model; E-H). When omission exists, the LAN-only approach underestimates response threshold a (A) and overestimates drift rate v (B). This bias emerges even when omission rate is lower than 5% but higher than 1% (C, D). With the LAN+OPN approach, both threshold a (E, G) and drift rate v (F, H) are properly recovered. Model recovery for DDM model with constant threshold (I). When omissions are ignored (LAN-only model), the posterior distribution of boundary collapse deviates from ground truth (zero) even when omission rates is less than 5%, while LAN+OPN model can properly recover the constant boundary. Error bars refer to 95% confidence intervals of mean posteriors across multiple synthetic data sets from the same parameter configuration. Gray lines represent identity lines.

(5) When it comes to collapsing thresholds models, I'm not sure that parameter recovery is the key interest of many researchers. In general, it's known that collapsing thresholds models don't have the best parameter recovery, and this gets extremely poor when considering a Weibull collapse (e.g., Evans et al., 2020; A parameter recovery assessment of time-variant models of decision-making). Instead, most research using collapsing thresholds models has focused on "model comparison", and determining

whether data appear to be more likely under a fixed thresholds or collapsing thresholds account. Importantly, I think this means that a parameter recovery assessment perhaps isn't the most useful one when including collapsing thresholds; a model recovery assessment is. Specifically, this would involve assessing whether data generated by fixed thresholds models is more likely to be better accounted for by a collapsing thresholds model when omissions are excluded (i.e., giving the fixed thresholds RT distribution a shape more typically associated with collapsing thresholds) vs when they are included. If this is the case, then the current manuscript could be a very important finding, and would suggest that previous work finding evidence for collapsing thresholds models with deadline data were potentially biased if they excluded omissions (including some of my own work).

Response:

Thanks for the comment and suggestion. As the reviewer suggested, we performed additional simulation:

1) Simulate DDM data with different deadline and thus with 1~10% omission rate
2) Fit DDM and ANGLE model with and without OPN and calculate WAIC
3) Examine the posterior distribution of boundary collapse and compare with the ground truth (no boundary collapse)

With this setup, we found that, LAN-only model does not strongly support a DDM with a linearly collapsing boundary (e.g., ANGLE model) when the omission rate is < 1%. However, as the omission rate increases (even when it is equal or less than 5%), the posterior sample of boundary collapse deviates from 0. By considering the omissions in the model, we found that DDM with constant boundary can be correctly identified from the data, even when omission rate is higher (~5%).

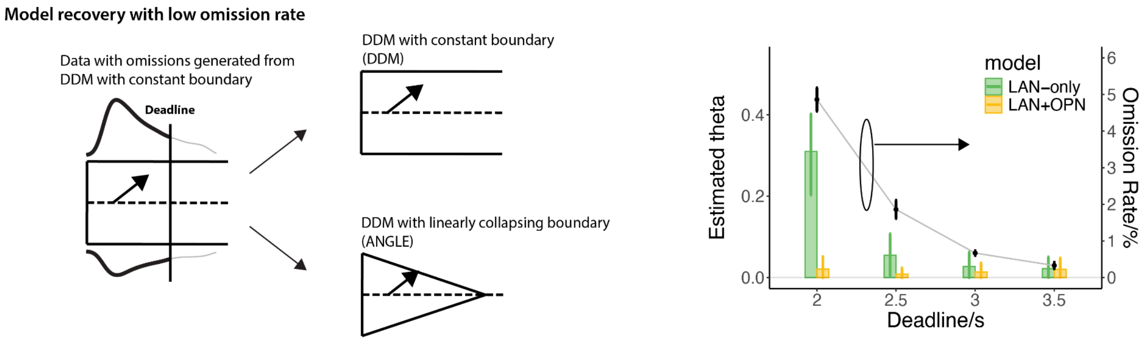

(6) In general, the details in the current manuscript are fairly lacking, particularly in terms of the simulation. For example, what scaling constant was used? Were ter and z fixed for generating values, or also fixed in terms of estimation? If fixed in terms of estimation, doesn't fixing ter (i) seem practically unreasonable, and (ii) have the potential to inflate the recovery performance of the collapsing thresholds model? Please try and make the process as reproducible as possible based on the details in the manuscript.

Response:

We thank the reviewer for pointing out this important issue in the methods part. We have included more details for the procedures in the methods part:

Training of LAN and OPN

The training and setup for LANs in the current paper is exactly the same as in the foundational methods paper (see Supporting Information for a brief summary; [38]). For OPN, we followed the same procedure with a difference in the training data structure: inputs of OPN include model parameters and deadline and each combination of input has one simulated omission rate value (Figure 1).

Simulation and inference

All models were simulated with a fixed unbiased starting point (coded as `z` = 0.5) and a fixed non-decision time (t). We generated 1000 trials based on the evidence accumulation process with a time step of 0.001s (see Table S2 in Supporting Information for details of model parameters). Trials with reaction time longer than a predefined deadline are masked. All models were fit via MCMC, where we estimated the posterior distributions using the No U-Turn Sampler (NUTS; [44]). We sampled 4 chains from the posterior distributions in each model with 1000 warmup samples followed by 1000 actual samples. In each model, we fixed the value of starting point bias and inferred non-decision time, drift rate, boundary parameters, and probability of lapse (see Table S3 in Supporting Information for details of prior distributions).

Reviewer #3: In decision-making experiments, it is frequent for participants to take very long to respond on some trials. These trials can disproportionately affect the interpretation of cognitive processes underlying decisions on other trials. To get around this problem, experimenters usually impose a response deadline and ignore trials, called omissions, where participants don't respond before the deadline. In this manuscript, Leng et al. show that this practice can be problematic. Using recently developed computational approximation methods of latent cognitive parameters, the authors show that ignoring omissions can lead to incorrect estimation of latent cognitive parameters. To overcome this hurdle, Leng et al. propose to combine the Latent Approximation Network (LAN) with a new Omission Probability Network (OPN). The authors have developed a Python package that can be used by the scientific community to correctly estimate cognitive parameters for sequential sampling models in circumstances where omissions are expected.

The paper is clearly written. The analysis is thorough and the authors show that the new tool is useful. I suspect the application of this tool will be for a very niche community. Nevertheless, it serves an important purpose for the community of mathematical psychologists studying sequential sampling models of decision-making.

I have only two recommendations for the improvement of the manuscript:

1. The authors show how omissions affect likelihood estimates made by LANs. They argue that even though these results are simulated using LANs, the results apply more broadly, even to cases where the analytical likelihoods are available, such as Drift Diffusion Models (pg 7, last paragraph). I think it will benefit the readers of this manuscript, and broaden the appeal of this paper, if the authors could show through a simple simulation where parameters of a DDM are being incorrectly estimated in an experiment (or perhaps even a set of experiments) where omission are ignored. The authors can re-analyse the data using their approach and work out the difference between existing estimates and the estimates obtained using their approach. This will also elucidate how much benefit this approach carries. Is it a very small change in the estimate (under normal experimental conditions) or a significant difference.

Response:

We thank the reviewer for this suggestion. We would like to clarify that LAN is only used for approximation of likelihood in the posterior sampling, and all datasets were generated by simulating the stochastic process (the cognitive process model, DDM etc.) faithfully. LANs played no role in the generation of the datasets themselves. In Figure 2, the data generation process is indeed the standard DDM with constant boundary, and we have included additional simulations with lower omission rate. We have updated the actual steps in the methods to make this clearer, and we hope this could convince the reviewer that our approach is suitable to a broader range of researchers.

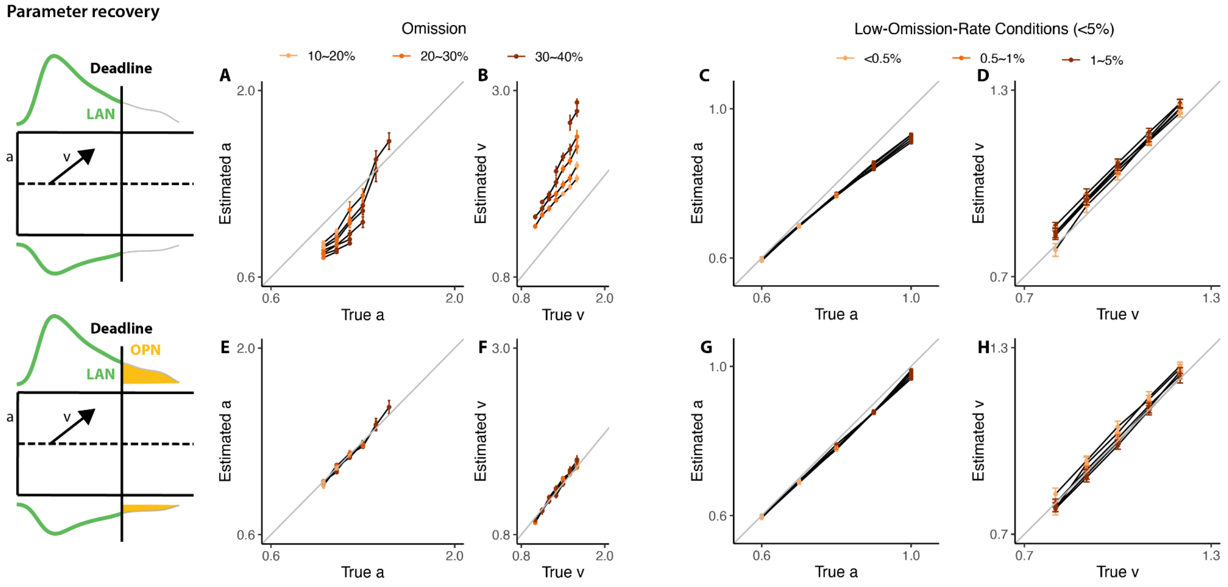

2. Could the authors provide a brief summary of Likelihood Approximation Networks, perhaps in Supporting Information? I looked up the previous references cited by the authors (refs 4, 23) but readers would be interested to know how LANs were trained in this case, how many parameters did they approximate, what is their parameter recovery like, etc.

Response:

We thank the reviewer for the comments regarding the missing details about how LAN and OPN are trained. As the reviewer mentioned, reference #23 is the original paper about LAN and the setup for LANs in the current paper is exactly the same as in the foundational methods paper.

We have added a line in the methods to explicitly state that the training and testing of LAN and OPN is fully aligned with reference #23.

> Training of LAN and OPN
>
> The training and setup for LANs in the current paper follow exactly the recipe in the foundational methods paper (see Supporting Information for a brief summary; [38]). For OPN, we followed the same procedure with a difference in the training data structure: inputs of OPN include model parameters and deadline and each combination of input has

one simulated omission rate value (Figure 1). No reaction time distributions need to be constructed for the omission probability outputs that serve OPN training.

For the convenience of readers, we have extracted and included relevant information in the Supporting Information (the citation number reflects the order in the Supporting Information).

The primary objective of LAN is to learn the function mapping from a generative model's parameter space and behavioral data (response time and choice) to its corresponding log-likelihood. Consequently, the inputs to a given MLP consist of the model parameter vector, response time (rt) and choice (c). The network's output is a scalar representing the predicted log-likelihood.

The LAN architectures are comprised of fully connected layers designed to balance computational efficiency with approximation accuracy. Depending on the complexity of the underlying generative model, the final architectures utilize between 3 and 5 hidden layers, with each layer containing 100 to 200 nodes. The hidden layers employ the hyperbolic tangent non-linear activation function, while the output layer consists of a single node with a linear activation function to yield the continuous log-likelihood estimate.

The LANs are trained on empirical likelihood datasets derived from simulations. Rather than using the standard mean squared error (MSE), the authors utilize the Huber loss function. This choice provides robust optimization because the Huber loss transitions from a quadratic penalty to a linear penalty for larger errors [7],making the training process significantly less sensitive to extreme outliers present in the empirical likelihood estimates.

The dataset is split into a training set (80%) and a validation set (20%) to monitor for overfitting. Optimization is performed using the Adam optimizer [8] with an initial learning rate of 0.001. To fine-tune convergence, a learning rate scheduling procedure reduces the learning rate by a factor of 10 (multiplied by 0.1) if the validation loss fails to decrease for 10 consecutive epochs. Training is regulated by an early stopping criterion, which terminates the process if the validation loss does not improve for 20 consecutive epochs or if the learning rate drops below a minimum threshold of 10^(-6). The networks were implemented in PyTorch [9].
