# Supplementary material for: The perils of omitting omissions when modeling evidence accumulation
Source: PLoS Comput Biol. 2026 Aug 21;22(8):e1014667. doi: 10.1371/journal.pcbi.1014667 (PMC13521452; doi:10.1371/journal.pcbi.1014667)
Supplement: Supplementary file 2 [file pcbi.1014667.s001.docx]

**Supporting Information**

1. **Samples of publications with response deadline and reported omission rate > 2%**

**Table A1. Samples of publications with response deadline and reported omission rate > 2%**

| **Publication** | **Deadline/ms** | **Mean OR/%** | **Range of OR across experiment conditions/%** | **Range of OR across**  **subjects/%** |
| --- | --- | --- | --- | --- |
| Pleskac, 2018 [1] | Study 1: 850  Study 2: 630 | Study1: 3  Study2: 10 | Study1: 1～5  Study2: 5~15 | Study1: 0~10  Study2: 1~38 |
| Grahek, 2022 [2] | 750 | 7 | 5~10 | 0~30 |
| Evans, 2020 [3] | Experiment 2: 1300 | 2.8 | 1~4.6 | 0~8  (Note: subjects with omission rate>10% were excluded) |
| Katsimpokis, 2020 [4] | Experiment 3:  683, 1350 or no deadline | For all conditions with deadline: 5.8 | 683 ms: 8.8~10.8  1350 ms: 0.8~2.6 | 683 ms: 2~28  1350 ms: 0~6.5 |
| Teoh, 2020 [5] | Study1:  1500 or 10000 | 2.5 | 1500 ms: 4.7  10000 ms: 0.3 | 1500 ms: 0~17  10000 ms: 0~2.5 |

2. **Summary of training procedure for LAN (adapted from the original paper about LAN** [6]**)**

The primary objective of LAN is to learn the function mapping from a generative model's parameter space and behavioral data (response time and choice) to its corresponding log-likelihood. Consequently, the inputs to a given MLP consist of the model parameter vector, response time (rt) and choice (c). The network's output is a scalar representing the predicted log-likelihood.

The LAN architectures are comprised of fully connected layers designed to balance computational efficiency with approximation accuracy. Depending on the complexity of the underlying generative model, the final architectures utilize between 3 and 5 hidden layers, with each layer containing 100 to 200 nodes. The hidden layers employ the hyperbolic tangent non-linear activation function, while the output layer consists of a single node with a linear activation function to yield the continuous log-likelihood estimate.

The LANs are trained on empirical likelihood datasets derived from simulations. Rather than using the standard mean squared error (MSE), the authors utilize the Huber loss function. This choice provides robust optimization because the Huber loss transitions from a quadratic penalty to a linear penalty for larger errors [7],making the training process significantly less sensitive to extreme outliers present in the empirical likelihood estimates.

The dataset is split into a training set (80%) and a validation set (20%) to monitor for overfitting. Optimization is performed using the Adam optimizer [8] with an initial learning rate of 0.001. To fine-tune convergence, a learning rate scheduling procedure reduces the learning rate by a factor of 10 (multiplied by 0.1) if the validation loss fails to decrease for 10 consecutive epochs. Training is regulated by an early stopping criterion, which terminates the process if the validation loss does not improve for 20 consecutive epochs or if the learning rate drops below a minimum threshold of ${10}^{-6}$. The networks were implemented in PyTorch [9].

3. **Additional information for range of parameters used for generation of data**

**Table A2. Parameters for dataset simulation**

| **DDM with constant boundary** | | **DDM with collapsing boundary** | | | |
| --- | --- | --- | --- | --- | --- |
|  | | **Linearly (ANGLE)** | | **Nonlinearly (WEIBULL)** | |
| v | High omission rate:1~1.6  Low omission rate: 0.8~1.2  Model recovery: 1 | v | 1.3  Omission source recovery: 1 | v | 1.3 |
| a | High omission rate:1~2  Low omission rate: 0.6~1  Model recovery: 0.9 | a | 1~2  Omission source recovery: 1 | a | 1~1.2 |
| deadline | High omission rate: 1.25 s  Low omission rate: 2.5 s |  | 1.25 |  | 1.25 |
| z | 0.5 | | | | |
| t | 0.3 s | | | | |
|  | | $\theta$ | 0.5~1  Omission source recovery: 0.25~0.8 | $\alpha$ | 0.5~1.5 |
|  |  |  |  | $\beta$ | 1~2 |

4. **Additional information for prior distribution used for Bayesian inference**

**Table A3. Prior distribution for Bayesian inference**

| **DDM with constant boundary** | | **DDM with collapsing boundary** | | | |
| --- | --- | --- | --- | --- | --- |
|  | | **Linear (ANGLE)** | | **Nonlinear (WEIBULL)** | |
| v | $\sim Uniform(-3,3)$ | | | | |
| a | $\sim Uniform(0.3,2.5)$ | | | | |
| z | 0.5 | | | | |
| t | $\sim Uniform(0,2)$ | | | | |
|  | | $\theta$ | $\sim Uniform(0,1.2)$ | $\alpha$ | $\sim Uniform(0.5,4.5)$ |
|  |  |  |  | $\beta$ | $\sim Uniform(0.5,4.5)$ |

5. **Parameter recovery for independent racing model**

To test whether our findings about DDM is specific to the selection of this model, we conducted additional simulations using the race model to investigate the impact of omitting omissions. In contrast to DDM, the race model has independent accumulator for each option. Without loss of generality, we considered race model with binary choices:

$dx_{t}^{i}=v^{i}dt+cdW, x_{0}^{i}=z_{i}*a;i=1,2$

where $x_{t}^{i}$ is the accumulated evidence for options $i$, $v^{i}$ is the drift rate for option $i$, and $z_{i}$ is the starting point bias for option $i$. A decision is made when accumulated evidence for one of the two options reaches threshold $a$. We also include non-decision time $t$ in the model. For the simplicity and demonstration focus, we fixed all starting point bias at 0.

Our results demonstrate that, similar to the DDM, omitting omissions leads to biased parameter estimates in race models even when omission rate is 5~10%, particularly concerning drift rates and decision thresholds, such that a LAN-only model will overestimate drift rate and underestimate threshold when (Fig A1). These findings confirm that accounting for omissions is critical across multiple sequential sampling models.

**Fig A1. Parameter recovery of race model with/without including omissions.** Across different levels of omission rate **(A)**, LAN-only model overestimates drift rate for both accumulators and underestimate threshold when omission rate is between 5~10 %, compared to the recovered posterior when no omissions exist (e.g., no stringent deadline). Additionally, the model tends to overestimate non-decision time. With the LAN+OPN model, the parameter recovery aligns with the cases when no omission exists.

6. **Additional simulation for parameter recovery of probability of lapse**

To confirm that the failure of lapse rate recovery is not due to how we set up the lapse distribution, we implemented additional simulations when no omission exists (Fig A2). We found that our LAN-only model can then recover the lapse rate when omission is absent. As we apply more stringent deadlines, the lapse rate is underestimated in the model not considering omissions.

**Fig A2. Recovery of lapse with and without the existence of omissions.** The omissions from evidence accumulation (EA; for this demonstration we used ANGLE model) and lapses increases with more stringent deadline **(A)**. Across different deadline and omission rates, LAN+OPN model can properly recover the lapse probability, while for LAN-only model the lapse probability can only be recovered when no omissions from lapses exist **(B)**.

7. **Parameter recovery with different deadlines (ANGLE model for demonstration)**

**Fig A3. Comparison between LAN-only and LAN+OPN approaches with different deadline values (using ANGLE model as example).** With either a tighter **(A-B)** or looser **(E-F)** deadline, LAN-only approach failed to properly recover boundary parameters (a: initial threshold,
$\theta$: boundary collapse), while the bias is larger in the tighter deadline conditions **(A-B)**. For both settings of deadline, LAN+OPN approach recovered the true boundary much better **(C-D; G-H)**. Error bars indicate 95% confidence intervals of the mean posterior. Gray lines represent identity lines.

References

1.	Pleskac TJ, Cesario J, Johnson DJ. How race affects evidence accumulation during the decision to shoot. Psychon Bull Rev. 2018;25: 1301–1330.

2.	Grahek I, Frömer R, Prater Fahey M, Shenhav A. Learning when effort matters: neural dynamics underlying updating and adaptation to changes in performance efficacy. Cereb Cortex. 2023;33: 2395–2411.

3.	Evans NJ, Hawkins GE, Brown SD. The role of passing time in decision-making. J Exp Psychol Learn Mem Cogn. 2020;46: 316–326.

4.	Katsimpokis D, Hawkins GE, van Maanen L. Not all speed-accuracy trade-off manipulations have the same psychological effect. Comput Brain Behav. 2020;3: 252–268.

5.	Teoh YY, Yao Z, Cunningham WA, Hutcherson CA. Attentional priorities drive effects of time pressure on altruistic choice. Nat Commun. 2020;11: 3534.

6.	Fengler A, Govindarajan LN, Chen T, Frank MJ. Likelihood approximation networks (LANs) for fast inference of simulation models in cognitive neuroscience. Elife. 2021;10. doi:10.7554/eLife.65074

7.	Huber PJ. Robust estimation of a location parameter. Springer Series in Statistics. New York, NY: Springer New York; 1992. pp. 492–518.

8.	Kingma DP, Ba J. Adam: A method for stochastic optimization. arXiv [cs.LG]. 2014. doi:10.48550/arXiv.1412.6980

9.	Paszke A, Gross S, Massa F, Lerer A, Bradbury J, Chanan G, et al. PyTorch: An Imperative Style, High-Performance Deep Learning Library. Advances in Neural Information Processing Systems. 2019;32.
