# Supplementary material for: The perils of omitting omissions when modeling evidence accumulation
Source: PLoS Comput Biol. 2026 Aug 21;22(8):e1014667. doi: 10.1371/journal.pcbi.1014667 (PMC13521452; doi:10.1371/journal.pcbi.1014667)
Supplement: Peer review history

# Peer review: Decision Letter 0

**DOI:** 10.1371/journal.pcbi.1014667.r001

**Authors:** Daniele Marinazzo, Iris Vilares

23 Feb 2026

The perils of omitting omissions when modeling evidence accumulation

PLOS Computational Biology

Dear Dr. Leng,

Thank you for submitting your manuscript to PLOS Computational Biology. After careful consideration, we feel that it has merit but does not fully meet PLOS Computational Biology's publication criteria as it currently stands. Therefore, we invite you to submit a revised version of the manuscript that addresses the points raised during the review process.

Please submit your revised manuscript by 5/31/2026. If you will need more time than this to complete your revisions, please reply to this message or contact the journal office at ploscompbiol@plos.org. When you're ready to submit your revision, log on to https://www.editorialmanager.com/pcompbiol/ and select the 'Submissions Needing Revision' folder to locate your manuscript file.

Please include the following items when submitting your revised manuscript:

* A letter that responds to each point raised by the editor and reviewer(s). You should upload this letter as a separate file labeled 'Response to Reviewers'. This file does not need to include responses to formatting updates and technical items listed in the 'Journal Requirements' section below.

* A marked-up copy of your manuscript that highlights changes made to the original version. You should upload this as a separate file labeled 'Revised Manuscript with Track Changes'.

* An unmarked version of your revised paper without tracked changes. You should upload this as a separate file labeled 'Manuscript'.

If you would like to make changes to your financial disclosure, competing interests statement, or data availability statement, please make these updates within the submission form at the time of resubmission. Guidelines for resubmitting your figure files are available below the reviewer comments at the end of this letter

We look forward to receiving your revised manuscript.

Kind regards,

Iris Vilares

Academic Editor

PLOS Computational Biology

Daniele Marinazzo

Section Editor

PLOS Computational Biology

**Journal Requirements:**

1) We ask that a manuscript source file is provided at Revision. Please upload your manuscript file as a .doc, .docx, .rtf or .tex. If you are providing a .tex file, please upload it under the item type u2018LaTeX Source Fileu2019 and leave your .pdf version as the item type u2018Manuscriptu2019.

2) Please upload all main figures as separate Figure files in .tif or .eps format. For more information about how to convert and format your figure files please see our guidelines:

https://journals.plos.org/ploscompbiol/s/figures

3) We have noticed that you have uploaded Supporting Information files, but you have not included a list of legends. Please add a full list of legends for your Supporting Information files after the references list.

**Reviewers' comments:**

Reviewer's Responses to Questions

**Comments to the Authors:**

**Please note here if the review is uploaded as an attachment.**

Reviewer #1: In The Perils of Omitting Omissions when Modeling Evidence Accumulation, the authors study the effect of ignoring missed trials on parameter estimation in diffusion decision models of decision making behavior. Using simulations, they demonstrate that ignoring omissions can lead to biased parameter estimates. They show that unbiased estimates can be obtained by incorporating the probability of response omission into the likelihood function. To enhance computational efficiency, they propose the use of likelihood approximation networks and omission probability networks.

Overall, the paper is well-written and, for the most part, easy to follow. The authors' argument that omissions should be included in the likelihood function is theoretically sound and convincing. However, I am less convinced about the practical relevance.

Specifically, my primary concern with the paper is the likelihood that current practices of omitting omissions significantly influence the literature. The authors do not provide empirical data on the actual omission rates reported in the literature. Their simulations show that higher omission rates lead to more biased parameter estimates, but these simulations use parameter settings that result in omission rates of 10% to 30%. Such rates are unusually high. For this review, I briefly examined some open-access datasets* that I am familiar with and found omission rates between 1% and 2%. It also makes sense that these rates are so low, since participants are normally penalized for missing responses in one way or another, and they adjust their behavior accordingly to ensure they respond in time. Even in paradigms that are explicitly designed to test omission rates, the reported omission rates are in the range of 2% to 6% (e.g., Damaso et al., 2022, 10.3758/s13421-021-01265-z ). Additionally, there already are methods to account for omission rates, and these have been applied in cases where omissions were hypothesized to potentially influence the results (e.g., Howard et al., 2020 10.3758/s13423-020-01741-8; Evans et al., 2018, 10.1111/cogs.12627 ).

This need not be the case in all studies, and perhaps my selection from the literature is just biased. But are there then studies where the omission rates are so high? Perhaps in clinical studies?

Additionally, in their initial simulations, the authors assume that omissions are solely the result of the accumulated evidence not reaching the threshold yet—referred to as task omissions in Damaso et al.’s terminology. However, as the authors acknowledge, this may not be the only or even the primary source of omissions. Other possibilities include attentional lapses and/or an intrinsic lack of evidence accumulation. While the authors address the attentional lapse hypothesis, I found the setup of this simulation difficult to understand and struggled to interpret the results. Specifically, why is the lapse rate not recovered at all with LAN-only models? Do the LAN models recover the lapse rate in the absence of omissions? The LAN-only panels in Figures B and D appear identical; is this correct?

More importantly, I believe the setup of this simulation overlooks a critical question. Given an empirical dataset, we do not know the source of omissions—it could be missed deadlines or attentional lapses. The current simulation assumes that omissions are primarily due to missed deadlines (even the 'high' lapse rate condition has a probability of only 0.05, compared to up to 30% omissions due to missed deadlines) and then demonstrates that the parameters can be recovered. However, can the presented methods also be used to determine whether the omissions are due to missed deadlines or attentional lapses?

Finally, I found the content of the paper highly focused on the DDM, while it is framed as a paper investigating SSMs more generally. How large is the influence of neglecting omissions in race models? Is there perhaps a stronger case to be made there?

Minor points

p. 3, line 87: “recent extensions of SSM models”. This is really very minor but the work on urgency has been around for a while now, I wouldn’t call it ‘recent’ anymore.

p. 3, line 89-93: The (critical) study on optimality of collapsing bounds by Boehm et al. (2020) should be mentioned here: 10.3758/s13414-019-01806-4

p. 5, line 143: The parametrisation of z and a should be clarified. If the bounds are at [-a, a], a start point of z=0 is unbiased. If the bounds are at [0, a], a start point of z=0.5*a is unbiased.

p. 5: Are between-trial variabilities in drift rates, start points, and non-decision times included in the simulations?

p. 10, line 245: “Larger than accurate bias parameters” – Maybe I am missing something, but Figure 2B doesn’t include bias (start point) parameters?

p. 18, line 362: Evans et al., (2019) should be included in the discussion of the recoverability of time-varying parameters such as collapsing bounds: 10.3758/s13428-019-01218-0

p. 18–20: I had difficulty understanding the rationale for including the non-linearly collapsing boundary case. As the authors acknowledge, parameter recovery for such models is very poor, even in the best-case scenario with no omissions. From what I understand, the authors suggest that, despite the inability to recover the parameters accurately, it might still be possible to recover the shape of the boundary. However, Figure 6E appears to show bounds that are very close to linear, although it is challenging to interpret the panel clearly.

*datasets/papers:

- Wagenmakers et al. (2008; 10.1016/j.jml.2007.04.006), experiment 1: 2% excluded, experiment 2: 1.5% excluded (range 300 – 2500 ms);

- Miletić & van Maanen (2019; 10.1016/j.cogpsych.2019.01.002 ) exp 1: deadline 1.5 seconds, in total 77 missed responses, out of 18868 trials (across all subjects)

- Forstmann et al. (2008; 10.1523/JNEUROSCI.1465-08.2008): Behavioral session, 1.5 seconds stimulus, on average 2% of trials excluded. One subject had 18% omissions – when excluding that participant, the average omission rate was 1.1%. These exclusions include fast guesses (<0.15 s response) as well as missed deadlines.

- Damaso et al., (2022) use an explicit distraction paradigm intended to study omissions, and even then they find only 6.4% omissions in elderly subjects, and 2.2% in young subjects.

Reviewer #2: The current manuscript assesses the impact of ignoring omissions (i.e., non-responses) in typical experimental data. Specifically, the current manuscript assesses the impact of ignoring omissions on the parameter recovery of different evidence accumulation models, where the generating process produces data that would result in omissions (i.e., response times longer than the response deadline). The current manuscript finds that parameter recovery is greatly impaired when these omissions are ignored, but is near perfect (at least, for regular diffusion models) when these omissions are explicitly included in the model.

I have mixed feelings about the current manuscript. On the one hand, I think it’s very interesting work, and the results make it clear that data with high omissions produce unreliable parameter estimates without the inclusion of omissions. On the other hand, I feel as though the contributions are somewhat overstated, and the simulations aren’t as thorough (and/or are lacking important details) as they could be. Therefore, I think that the current manuscript will make a nice contribution to the literature, but requires some revision both in terms of the literature discussed and the scope of the simulations, which I detail below.

(1) The idea of omissions is something that, in my opinion, has had a decent amount of research put into it, and has been addressed in evidence accumulation models previously. However, the current manuscript ignores a lot of the existing literature, indirectly overstating its contribution. For example, I find it crazy that the seminal paper on omissions in response time data, which the current manuscript indirectly utilises, isn’t even cited (Ulrich & Miller, 1994; Effects of truncation on reaction time analysis). Personally, I’ve modelled omissions in evidence accumulation models in several situations, and the idea of modelling omissions forms the basis for my double responding models (e.g., Evans et al., 2020; Double responding: A new constraint for models of speeded decision making). Furthermore, the lapse process seems to just be the standard Ratcliff contamination process, which I think should be more prominently noted (and that this isn’t the only way to think about “lapses”, e.g., see Guy Hawkins’ work on mind wandering). I’m only naming the first things that come into my head, but there’s a lot more usage of omissions modelling than the current manuscript currently indicates, and I think that the authors need to be much more thorough in their discussion of the literature in a revision.

(2) I think that the description of the omissions process is made much more complex than it needs to be, and the computational burden of implementing this using standard approaches is overstated. In truth, the probability of an omission occurring is just the survivor function of the accumulation process (i.e., 1 – CDF_1 – CDF_2 … – CDF_n), and this doesn’t add too great of a computational burden for analytic models (e.g., see Evans et al., 2018; Modeling the covariance structure of complex datasets using cognitive models: An application to individual differences and the heritability of cognitive ability; or see Howard et al., 2020; How is multi-tasking different from increased difficulty?) or for simulation-based methods (e.g., see Evans et al., 2020; Double responding: A new constraint for models of speeded decision making). I agree that it adds a problematic computational burden for numeric methods for calculating the likelihood (i.e., if you already require integral estimation for getting the PDF, then getting the CDF is quite problematic), but I think these situations are becoming more rare (e.g., I used a numeric method for collapsing thresholds models with deadline data in Evans et al., 2020; The role of passing time in decision-making; which made modelling omissions too computationally taxing, but I think most people would use simulation-based methods these days, as they’re faster). I still think the current framework is useful, but I disagree with the argument that the omissions method cannot be (or is too computationally taxing to) implement in existing frameworks.

(3) I disagree with the general idea that response deadlines are commonly used to incentivize speedy responding. In general, when it comes to experimental psychology, I think the most common method to incentivize speedy responding are the classic SAT emphasis instructions (i.e., just telling people to focus on speed or accuracy), and deadlines are usually avoided for the censoring/omissions reasons discussed in Ulrich & Miller (1994) and in the current manuscript (unless deadlines are specifically of interest). I know the use of deadlines are more common in other areas, though (e.g., I think this is the case in decision neuroscience?), so perhaps it would be good to be more specific in discussing what areas typically use response deadlines (and maybe including more citations to usages).

(4) Building on the last point, while some type of censoring is typically used in response time tasks, either through deadlines or excluding “long” responses that are thought to be contaminants (e.g., the participant lost attention), the censoring is usually much later in time (e.g., 5s), and typically excludes relatively fewer trials than is the case in these simulations. While the supplementary materials do provide recoveries using a slightly different deadlines (1s or 1.5s, vs the 1.25s in the main text), this is only for the linear collapsing threshold model. Importantly, I would be much more interested to see how the standard diffusion model (i.e., the most used model for parameter estimation in real data) performs with much fewer omissions (and preferably, a much longer response deadline), as I think this is crucial for claims like “We found that parameter recovery was severely impacted when ignoring omissions at inference, even when omissions are relatively rare (5% of trials or fewer).”

(5) When it comes to collapsing thresholds models, I’m not sure that parameter recovery is the key interest of many researchers. In general, it’s known that collapsing thresholds models don’t have the best parameter recovery, and this gets extremely poor when considering a Weibull collapse (e.g., Evans et al., 2020; A parameter recovery assessment of time-variant models of decision-making). Instead, most research using collapsing thresholds models has focused on “model comparison”, and determining whether data appear to be more likely under a fixed thresholds or collapsing thresholds account. Importantly, I think this means that a parameter recovery assessment perhaps isn’t the most useful one when including collapsing thresholds; a model recovery assessment is. Specifically, this would involve assessing whether data generated by fixed thresholds models is more likely to be better accounted for by a collapsing thresholds model when omissions are excluded (i.e., giving the fixed thresholds RT distribution a shape more typically associated with collapsing thresholds) vs when they are included. If this is the case, then the current manuscript could be a very important finding, and would suggest that previous work finding evidence for collapsing thresholds models with deadline data were potentially biased if they excluded omissions (including some of my own work).

(6) In general, the details in the current manuscript are fairly lacking, particularly in terms of the simulation. For example, what scaling constant was used? Were ter and z fixed for generating values, or also fixed in terms of estimation? If fixed in terms of estimation, doesn’t fixing ter (i) seem practically unreasonable, and (ii) have the potential to inflate the recovery performance of the collapsing thresholds model? Please try and make the process as reproducible as possible based on the details in the manuscript.

Nathan J. Evans

Reviewer #3: In decision-making experiments, it is frequent for participants to take very long to respond on some trials. These trials can disproportionately affect the interpretation of cognitive processes underlying decisions on other trials. To get around this problem, experimenters usually impose a response deadline and ignore trials, called omissions, where participants don't respond before the deadline. In this manuscript, Leng et al. show that this practice can be problematic. Using recently developed computational approximation methods of latent cognitive parameters, the authors show that ignoring omissions can lead to incorrect estimation of latent cognitive parameters. To overcome this hurdle, Leng et al. propose to combine the Latent Approximation Network (LAN) with a new Omission Probability Network (OPN). The authors have developed a Python package that can be used by the scientific community to correctly estimate cognitive parameters for sequential sampling models in circumstances where omissions are expected.

The paper is clearly written. The analysis is thorough and the authors show that the new tool is useful. I suspect the application of this tool will be for a very niche community. Nevertheless, it serves an important purpose for the community of mathematical psychologists studying sequential sampling models of decision-making.

I have only two recommendations for the improvement of the manuscript:

1. The authors show how omissions affect likelihood estimates made by LANs. They argue that even though these results are simulated using LANs, the results apply more broadly, even to cases where the analytical likelihoods are available, such as Drift Diffusion Models (pg 7, last paragraph). I think it will benefit the readers of this manuscript, and broaden the appeal of this paper, if the authors could show through a simple simulation where parameters of a DDM are being incorrectly estimated in an experiment (or perhaps even a set of experiments) where omission are ignored. The authors can re-analyse the data using their approach and work out the difference between existing estimates and the estimates obtained using their approach. This will also elucidate how much benefit this approach carries. Is it a very small change in the estimate (under normal experimental conditions) or a significant difference.

2. Could the authors provide a brief summary of Likelihood Approximation Networks, perhaps in Supplementary Materials? I looked up the previous references cited by the authors (refs 4, 23) but readers would be interested to know how LANs were trained in this case, how many parameters did they approximate, what is their parameter recovery like, etc.

Some minor corrections:

l168: exist -> exists

l197-l198: "Hence, we train..." This sentence is ungrammatical. Perhaps remove 'that will'?

l396-l397: I had trouble finding the reference (ref 29) which provides the HSSM toolbox mentioned in this manuscript. I was able to find the toolbox on one of the author's github page. I would recommend either correcting the reference or publishing the github link for the package.

**********

**Have the authors made all data and (if applicable) computational code underlying the findings in their manuscript fully available?**

The PLOS Data policy requires authors to make all data and code underlying the findings described in their manuscript fully available without restriction, with rare exception (please refer to the Data Availability Statement in the manuscript PDF file). The data and code should be provided as part of the manuscript or its supporting information, or deposited to a public repository. For example, in addition to summary statistics, the data points behind means, medians and variance measures should be available. If there are restrictions on publicly sharing data or code —e.g. participant privacy or use of data from a third party—those must be specified.

Reviewer #1: Yes

Reviewer #2: None

Reviewer #3: Yes

**********

PLOS authors have the option to publish the peer review history of their article (what does this mean?). If published, this will include your full peer review and any attached files.

If you choose “no”, your identity will remain anonymous but your review may still be made public.

**Do you want your identity to be public for this peer review?**  For information about this choice, including consent withdrawal, please see our Privacy Policy.

Reviewer #1: No

Reviewer #2: No

Reviewer #3: No

[NOTE: If reviewer comments were submitted as an attachment file, they will be attached to this email and accessible via the submission site. Please log into your account, locate the manuscript record, and check for the action link "View Attachments". If this link does not appear, there are no attachment files.]

**Figure resubmission:**

**Reproducibility:**

?>

# Author comment: Author response to Decision Letter 1

**DOI:** 10.1371/journal.pcbi.1014667.r002

8 Jul 2026

Attachment Submitted filename: response to reviewers.pdf

# Peer review: Decision Letter 1

**DOI:** 10.1371/journal.pcbi.1014667.r003

**Authors:** Daniele Marinazzo, Iris Vilares

4 Aug 2026

Dear Dr. Leng,

We are pleased to inform you that your manuscript 'The Perils of Omitting Omissions when Modeling Evidence Accumulation' has been provisionally accepted for publication in PLOS Computational Biology.

Before your manuscript can be formally accepted you will need to complete some formatting changes, which you will receive in a follow up email. A member of our team will be in touch with a set of requests.

Please note that your manuscript will not be scheduled for publication until you have made the required changes, so a swift response is appreciated.

IMPORTANT: The editorial review process is now complete. PLOS will only permit corrections to spelling, formatting or significant scientific errors from this point onwards. Requests for major changes, or any which affect the scientific understanding of your work, will cause delays to the publication date of your manuscript.

Should you, your institution's press office or the journal office choose to press release your paper, you will automatically be opted out of early publication. We ask that you notify us now if you or your institution is planning to press release the article. All press must be co-ordinated with PLOS.

Thank you again for supporting Open Access publishing; we are looking forward to publishing your work in PLOS Computational Biology.

Best regards,

Iris Vilares

Academic Editor

PLOS Computational Biology

Daniele Marinazzo

Section Editor

PLOS Computational Biology

***********************************************************

Dear Dr. Leng,

Thank you for your revised manuscript and thorough reply to reviewers. Unfortunately, when re-inviting the same reviewers, only 1 of the 3 accepted to re-review the paper. However, based on the reviewer's feedback after rereading the paper and my own assessment, I think you adequately addressed all the reviewers' comments, and therefore the paper is acceptable for publication in PLoS CB. I would just suggest that you update your code and simulated data on GitHub to also include the new analyses and simulations you performed when replying to the reviewers (in case you haven't done so already).

Congratulations on the nice paper,

Iris Vilares

Reviewer's Responses to Questions

**Comments to the Authors:**

**Please note here if the review is uploaded as an attachment.**

Reviewer #2: I think that the authors have been extremely thorough in addressing my comments in the revised manuscript. The revised manuscript is much clearer in terms of contribution, and much more robust in terms of the conclusions with the additions of new simulations. I would recommend acceptance for publication.

Nathan J. Evans

**********

**Have the authors made all data and (if applicable) computational code underlying the findings in their manuscript fully available?**

The PLOS Data policy requires authors to make all data and code underlying the findings described in their manuscript fully available without restriction, with rare exception (please refer to the Data Availability Statement in the manuscript PDF file). The data and code should be provided as part of the manuscript or its supporting information, or deposited to a public repository. For example, in addition to summary statistics, the data points behind means, medians and variance measures should be available. If there are restrictions on publicly sharing data or code —e.g. participant privacy or use of data from a third party—those must be specified.

Reviewer #2: None

**********

PLOS authors have the option to publish the peer review history of their article (what does this mean?). If published, this will include your full peer review and any attached files.

If you choose “no”, your identity will remain anonymous but your review may still be made public.

**Do you want your identity to be public for this peer review?**  For information about this choice, including consent withdrawal, please see our Privacy Policy.

Reviewer #2: No

# Editor report: Acceptance letter

**DOI:** 10.1371/journal.pcbi.1014667.r004

**Authors:** Daniele Marinazzo, Iris Vilares

PCOMPBIOL-D-25-02343R1

The Perils of Omitting Omissions when Modeling Evidence Accumulation

Dear Dr Leng,

I am pleased to inform you that your manuscript has been formally accepted for publication in PLOS Computational Biology. Your manuscript is now with our production department and you will be notified of the publication date in due course.

The corresponding author will soon be receiving a typeset proof for review, to ensure errors have not been introduced during production. Please review the PDF proof of your manuscript carefully, as this is the last chance to correct any errors. Please note that major changes, or those which affect the scientific understanding of the work, will likely cause delays to the publication date of your manuscript.

Soon after your final files are uploaded, unless you have opted out, the early version of your manuscript will be published online. The date of the early version will be your article's publication date. The final article will be published to the same URL, and all versions of the paper will be accessible to readers.

For Research, Software, and Methods articles, you will receive an invoice from PLOS for your publication fee after your manuscript has reached the completed accept phase. If you receive an email requesting payment before acceptance or for any other service, this may be a phishing scheme. Learn how to identify phishing emails and protect your accounts at https://explore.plos.org/phishing.

Thank you again for supporting PLOS Computational Biology and open-access publishing. We are looking forward to publishing your work!

With kind regards,

Janani Seenivasan

PLOS Computational Biology | Carlyle House, Carlyle Road, Cambridge CB4 3DN | United Kingdom ploscompbiol@plos.org | Phone +44 (0) 1223-442824 | ploscompbiol.org | @PLOSCompBiol
